# Feeder-Free Generation and Long-Term Culture of Human Induced Pluripotent Stem Cells Using Pericellular Matrix of Decidua Derived Mesenchymal Cells

**DOI:** 10.1371/journal.pone.0055226

**Published:** 2013-01-31

**Authors:** Hayato Fukusumi, Tomoko Shofuda, Daisuke Kanematsu, Atsuyo Yamamoto, Hiroshi Suemizu, Masato Nakamura, Mami Yamasaki, Masatoshi Ohgushi, Yoshiki Sasai, Yonehiro Kanemura

**Affiliations:** 1 Division of Regenerative Medicine, Institute for Clinical Research, Osaka National Hospital, National Hospital Organization, Osaka, Japan; 2 Division of Stem Cell Research, Institute for Clinical Research, Osaka National Hospital, National Hospital Organization, Osaka, Japan; 3 Division of Molecular Medicine, Institute for Clinical Research, Osaka National Hospital, National Hospital Organization, Osaka, Japan; 4 Department of Neurosurgery, Osaka National Hospital, National Hospital Organization, Osaka, Japan; 5 Biomedical Research Department, Central Institute for Experimental Animals, Kawasaki, Kanagawa, Japan; 6 Department of Pathology and Regenerative Medicine, Tokai University School of Medicine, Isehara, Kanagawa, Japan; 7 Unit for Human Stem Cell Technology, Center for Developmental Biology, RIKEN, Kobe, Hyogo, Japan; 8 Organogenesis and Neurogenesis Group, Center for Developmental Biology, RIKEN, Kobe, Hyogo, Japan; 9 Department of Pediatric Neurosurgery, Takatsuki General Hospital, Takatsuki, Osaka, Japan; University of Udine, Italy

## Abstract

Human ES cells (hESCs) and human induced pluripotent stem cells (hiPSCs) are usually generated and maintained on living feeder cells like mouse embryonic fibroblasts or on a cell-free substrate like Matrigel. For clinical applications, a quality-controlled, xenobiotic-free culture system is required to minimize risks from contaminating animal-derived pathogens and immunogens. We previously reported that the pericellular matrix of decidua-derived mesenchymal cells (PCM-DM) is an ideal human-derived substrate on which to maintain hiPSCs/hESCs. In this study, we examined whether PCM-DM could be used for the generation and long-term stable maintenance of hiPSCs. Decidua-derived mesenchymal cells (DMCs) were reprogrammed by the retroviral transduction of four factors (OCT4, SOX2, KLF4, c-MYC) and cultured on PCM-DM. The established hiPSC clones expressed alkaline phosphatase, hESC-specific genes and cell-surface markers, and differentiated into three germ layers in vitro and in vivo. At over 20 passages, the hiPSCs cultured on PCM-DM held the same cellular properties with genome integrity as those at early passages. Global gene expression analysis showed that the GDF3, FGF4, UTF1, and XIST expression levels varied during culture, and GATA6 was highly expressed under our culture conditions; however, these gene expressions did not affect the cells’ pluripotency. PCM-DM can be conveniently prepared from DMCs, which have a high proliferative potential. Our findings indicate that PCM-DM is a versatile and practical human-derived substrate that can be used for the feeder-cell-free generation and long-term stable maintenance of hiPSCs.

## Introduction

Induced pluripotent stem cells (iPSCs) are generated from various somatic cells by introducing defined transcription factors [1], [2], and they have properties similar to those of embryonic stem cells (ESCs). iPSCs are expected to contribute greatly not only to the realization of regenerative medicine but also to understanding the molecular pathogenesis of many currently intractable diseases. The promise of cell-based therapies using human iPSCs (hiPSCs) is generally recognized, and has driven an intense search for good cell sources, reprogramming methods, and cell culture systems. However, their clinical application has yet to be realized.

In general, hiPSCs/human ESCs (hESCs) are generated and maintained on living feeder cells, such as mouse embryonic fibroblasts (MEFs) [2]–[4] or SNL cells [1], [5], or on a feeder-free culture substrate such as Matrigel [6]–[9], fibronectin [10]–[13], or human recombinant laminin-511 [14], [15]. For clinical applications, quality-controlled xenobiotic-free culture systems are required to minimize health risks from animal-derived pathogens and immunogens [16], [17]. Therefore, the use of primary human-derived living cells, like fibroblasts [18]–[21] or amnion-derived cells [22], is a hopeful approach, although some difficulties with these methods must still be overcome. We previously reported that the pericellular matrix of decidua-derived mesenchymal cells (PCM-DM) is an ideal human-derived material for maintaining hiPSCs/hESCs [23]. The maintenance activity of PCM-DM is similar to that of Matrigel, and its preparation is easy and reproducible, because decidua-derived mesenchymal cells (DMCs) can be obtained and expanded in large quantity [23].

In this study, we examined whether PCM-DM could be used for the feeder-free generation of hiPSCs and whether PCM-DM could maintain the cellular properties of hiPSCs over many passages. DMCs were reprogrammed by the retroviral transduction of four factors (OCT4, SOX2, KLF4, and c-MYC; i.e., OSKM) and cultured on PCM-DM. The hiPSCs established on PCM-DM (hiPSC-PCMDM) expressed alkaline phosphatase (ALP) activity and hESC-specific genes and surface markers, and they differentiated into all three germ layers in vitro and in vivo. After over 20 passages, the cellular properties were similar to those of the cells assayed at early passages and had genomic integrity. Global gene expression analysis showed that the expression levels of GDF3, FGF4, UTF1, and XIST varied during culture and GATA6 expression was high under our conditions; the expression of these genes did not affect pluripotency. These findings suggest that PCM-DM is a practical, human-derived substrate that can be used for both the generation and stable maintenance of hiPSCs.

## Materials and Methods

### Human Tissue and Cells

This study was carried out in accordance with the principles of the Helsinki Declaration, and approval to use human tissues was obtained from the ethical committee of Osaka National Hospital. The donor bloods were serologically tested for HBs, HCV, HIV, and syphilis. Full-term placental tissues and results of donor blood tests were collected at the Osaka National Hospital with written informed consent.

Human ESCs (clone KhES1) were obtained from Kyoto University (Kyoto, Japan) [24] and propagated at the Center for Developmental Biology, RIKEN, in accordance with Japanese guidelines on the utilization of human ES cells, under approval from the Ministry of Education, Culture, Sports, Science and Technology (MEXT) of Japan. Human iPS cells (clone 201B7) [1] were obtained from the RIKEN cell bank (Tsukuba, Japan) and propagated on mitomycin C-treated SNL feeder cells in Primate ES cell medium (ReproCELL, Kanagawa Japan) with basic fibroblast growth factor (bFGF; 4 ng/ml; R&D Systems, Minneapolis MN) or mitomycin C-treated mouse embryonic fibroblast (MEF) feeder cells in DMEM/F-12 (1∶1)-based culture medium, with 20% KnockOut SR (KSR, Invitrogen)/non-essential amino acids (0.1 mM, Invitrogen)/L-glutamine (2 mM, Invitrogen)/2-Mercaptoethanol (0.1 mM; Invitrogen)/bFGF (5 ng/ml; Wako Pure Chemical Industries, Ltd, Osaka Japan)/antibiotic-antimycotic, as previously described (hESC medium) [2], [25].

### Preparation of PCM-DM

Human DMCs were propagated from human term decidua vera in the DMEM/F-12 (1∶1)-based culture medium supplemented with 10% fetal bovine serum (FBS), HEPES (15 mM), and antibiotic-antimycotic (Invitrogen) at 37°C in 5% CO_2_ as previously described [23], [26]. PCM-DM was prepared as described previously [23]. Briefly, DMCs were seeded at 3.5×10^4^ cells/cm^2^ on gelatin-precoated culture plates in the culture medium at 37°C in 5% CO_2_. After three days in culture, the cells were rinsed with PBS once and treated with deoxycholate solution (0.5% sodium deoxycholate in 10 mM Tris-HCl, pH 8.0) at 4°C for 30 min. The treated plates were then washed six times with PBS by pipetting to flush off the cell debris, and stored under semi-dry conditions at 4°C.

### Generation and Propagation of hiPSCs on PCM-DM

**Figure 1:**
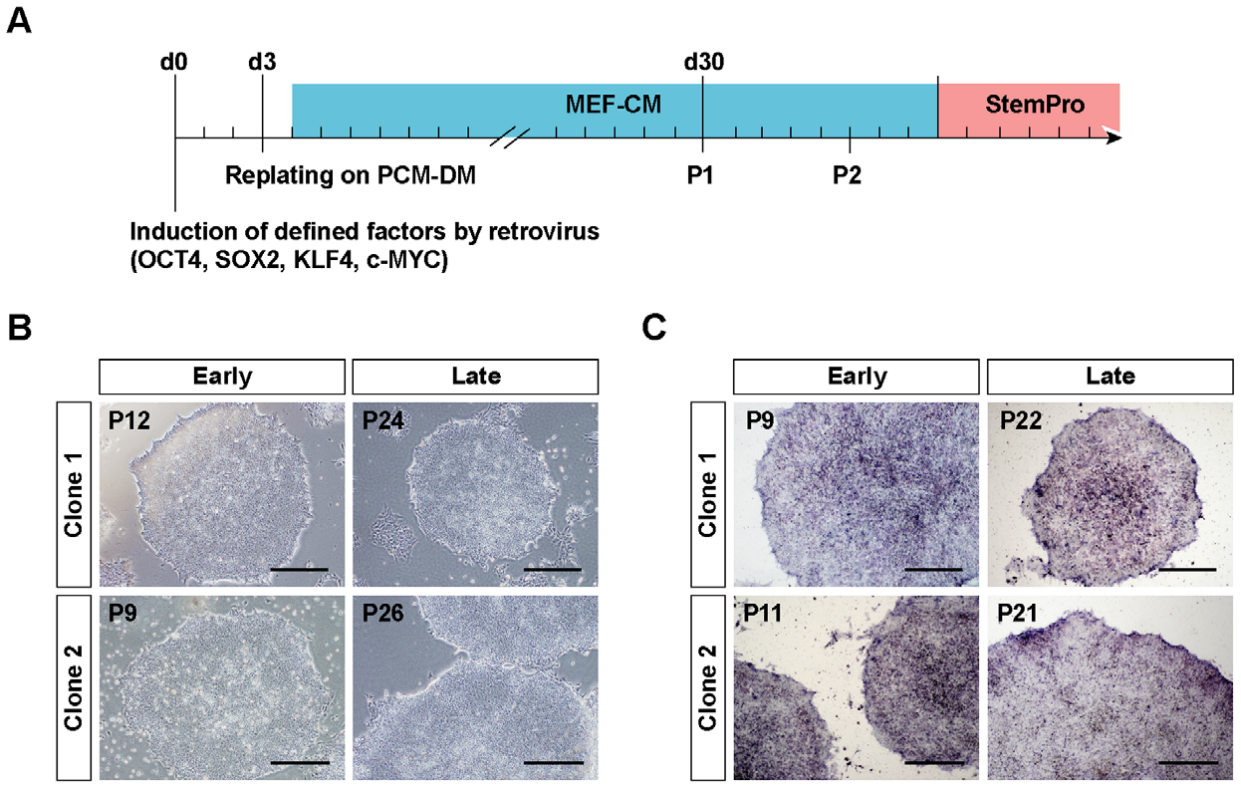
Generation of hiPSCs on PCM-DM. A) Schematic diagram for generating hiPSCs on PCM-DM. B) Representative morphology of the hiPSCs on PCM-DM at early and late passages. C) Alkaline phosphatase (ALP) staining of hiPSCs generated on PCM-DM. P, passage number. Scale bar = 500 µm.

Four pMXs retroviral vectors encoding four reprogramming factors (OSKM) were obtained from Addgene, Inc (Cambridge, MA) [1], and amphotropic retroviruses were produced by the transfection of Platinum-A retroviral packaging cells (Cell Biolabs Inc, San Diego, CA) using FuGENE® 6 Transfection Reagent (Roche Diagnostics, Indianapolis, IN). DMCs were infected with the retrovirus supernatants supplemented with polybrene (4 µg/ml, Nacalai, Kyoto, Japan). Three days post-infection, the cells were plated on PCM-DM at 2×10^4^ cells/60-mm-diameter culture dish, and the culture medium was exchanged the next day with MEF-conditioned medium (MEF-CM), prepared as described previously [6]. Briefly, mitomycin C-treated MEF cells were cultured on gelatin-coated plates in hESC medium consisting of DMEM/F-12 (1∶1)-based culture medium with 20% KnockOut SR (KSR, Invitrogen)/non-essential amino acids (0.1 mM, Invitrogen)/L-glutamine (2 mM, Invitrogen)/2-Mercaptoethanol (0.1 mM; Invitrogen)/bFGF (5 ng/ml; Wako Pure Chemical Industries, Ltd, Osaka Japan)/antibiotic-antimycotic for 24 hours at 37°C in 5% CO_2_. The supernatant was then collected and supplemented with an additional 5 ng/ml bFGF. The medium was changed daily until hESC-like colonies appeared. After 30 days of induction, the hESC-like colonies were picked up and replated on PCM-DM in MEF-CM (passage 1). After passage 2, the established hiPSCs were cultured with STEM-PRO hESC SFM (StemPro, Invitrogen) on PCM-DM (Fig. 1A).

Human iPSCs (201B7) propagated on SNL feeder cells in hESC medium were also cultured on PCM-DM in MEF-CM or StemPro medium.

### Cell Reprogramming Efficiency Using PCM-DM Versus Other Substrates

Six different DMC lines (DMC71, DMC72, DMC75, DMC76, DMC85 and DMC92) were reprogrammed under seven different culture conditions (6×10^4^ cells per culture), as follows: plated on MEFs with non-conditioned (NC)-hESC medium (control), on PCM-DM with MEF-CM, on Matrigel with MEF-CM, on gelatin with MEF-CM (autologous feeder) [27], on PCM-DM with NC-hESC medium, on Matrigel with NC-hESC medium, and on gelatin with NC-hESC medium. Cells reprogrammed using PCM-DM with NC-hESC medium were propagated further for detailed analyses.

### ALP Staining

Cells were fixed with 10% Formalin/PBS for 2 min. The fixed cells were washed with PBS once and then stained with 1-Step NBT/BCIP (Thermo SCIENTIFIC, Rockford, IL) for 30 min at room temperature (RT), according to the manufacturer’s specification.

### Quantitative Reverse Transcription-polymerase Chain Reaction (qRT-PCR)

Total RNA was extracted as described in the RNeasy Mini Kit (Qiagen, Valencia, CA), and cDNA was synthesized from 1 µg total RNA using random hexamers for reverse transcription, according to the manufacturer’s specification (SuperScript First-Strand Synthesis System for RT-PCR, Invitrogen). Quantitative PCR analysis was performed using gene-specific primers (Supplemental Table S1) with Power SYBR® Green PCR Master Mix, the 7300 real-time PCR system (Applied Biosystems, Foster, CA), and the comparative Ct method or standard curve method [28].

### Flow Cytometry (FCM) Analysis

Cells were pre-incubated with Y-27632 (Wako Pure Chemical Industries) for 30 min and then dissociated by trypsin/EDTA (Invitrogen). The dissociated cells were fixed with 4% paraformaldehyde (PFA) for 20 min on ice, washed with PBS, and then reacted with the following primary antibodies for 30 min at 4°C: anti-SSEA-4 (MC-813-70, Chemicon International Inc., Temecula, CA), anti-SSEA-3 (MC-631, Chemicon), anti-TRA-1-60 (TRA-1-60, Chemicon), anti-TRA-1-81 (TRA-1-81, Chemicon). After being washed, the cells were incubated with the appropriate secondary antibodies (Alexa-488-conjugated anti-mouse IgG, anti-mouse IgM, or anti-rat IgM, Molecular Probes, Eugene, OR) for 30 min at 4°C. The stained samples were analyzed by FACS Calibur flow cytometer (BD Biosciences, San Jose, CA).

### Microarray Analysis

Total RNA (100 ng) was used to synthesize aRNA using the 3′ IVT Express Kit, according to the manufacturer’s instructions (Affymetrix Inc., Santa Clara, CA). After aRNA purification, 15 µg of aRNA was fragmented and hybridized with a pre-equilibrated GeneChip array (Human Genome U133 Plus 2.0 gene expression array, Affymetrix) at 45°C for 16 hours. The GeneChip array was then washed, stained, and scanned according to the manufacturer’s instructions. The gene expression data were extracted using Affymetrix Expression Console software, and the Pearson’s coefficient of correlation was calculated for each pair of samples. Hierarchical cluster analysis was carried out using the “Manhattan” method for the similarity measurement and the “complete” method for linkage. These analyses were performed using GeneSpring GX software (Agilent Technologies, Inc., Santa Clara, CA). The data discussed in this publication have been deposited in NCBI's Gene Expression Omnibus [29] and are accessible through GEO Series accession number GSE37842 (http://www.ncbi.nlm.nih.gov/geo/query/acc.cgi?acc=GSE37842).

### Karyotype Analysis

Cells were cultured with colcemid (0.06 µg/ml; Invitrogen) at 37°C for 4 hours, and single-cell suspensions were prepared by 0.05% trypsin/EDTA dissociation after pre-treatment with Y-27632 to prevent apoptosis. After incubation in 75 mM KCl for 20 min, the cells were fixed in Carnoy fluid. The fixed samples were heat-denatured at 95°C for 2 hours, incubated in 0.025% trypsin for 10 sec, and stained with Giemsa (Merck, Darmstadt, Germany) for 7 min. The samples were observed under a microscope (Carl Zeiss), and metaphase samples were analyzed using Ikaros (MetaSystems, Altlussheim, Germany).

### Genotyping of Short Tandem Repeat (STR) Polymorphisms

Genomic DNA was extracted by DNAzol Reagent (Invitrogen). STR loci were investigated with the Powerplex 16 system (Promega, Madison, WI) using an ABI PRISM 3100 Genetic Analyzer (Applied Biosystems) and analyzed by GeneMapper software (Applied Biosystems) following the manufacturer’s instructions [26].

### Bisulfite Modification and DNA Sequencing

Genomic DNAs were bisulfite-treated with the EZ DNA methylation-Gold Kit (ZYMO Research, Orange, CA), according to the manufacturer’s instructions. The human OCT4 and NANOG promoter regions were amplified with specific primer sets (Supplemental Table S2) by TaKaRa Taq™ Hot Start Version (Takara Bio INC., Shiga, Japan). The PCR products were sub-cloned into the p2.1 vector (Invitrogen). Ten clones were sequenced with a universal primer by ABI PRISM 3100 Genetic Analyzer (Applied Biosystems) and analyzed by Sequence analysis software (Applied Biosystems), following the manufacturer’s instructions.

### In vitro Differentiation Assay

Cells were incubated with collagenase type IV (1 mg/ml; Invitrogen) for 3 min at 37°C and harvested by scraper. The harvested colonies were then incubated on petri dishes in hESC medium without bFGF to form embryoid bodies (EBs). After 8 days of floating culture, the EBs were harvested for transcript analysis. Some of the EBs were transferred to gelatin-coated chamber slides (Nunc, Roskilde, Denmark) and cultured for another week (total 15 days) in DMEM supplemented with 10% FBS, at 37°C in 5% CO_2_ [1].

### Teratoma Formation Assay

NOG (NOD/Shi-scid IL2Rgnull) mice [30] aged 6–8 weeks were maintained under specific-pathogen-free conditions in the Animal Facility of the Central Institute for Experimental Animals (CIEA). All mice studies were carried out in accordance with the guidelines of the Animal Care Committee at CIEA and approved by the Animal Care Committee at CIEA. The recipient mice were anesthetized by isoflurane inhalation (Dainippon Pharmaceutical Co., Ltd., Osaka Japan). Human iPSCs were xeno-transplanted into both subcutaneous tissues and kidney capsules. For subcutaneous transplantation, hiPSCs (1×10^6^ cells/0.1 ml of serum-free medium) were subcutaneously inoculated into the flank. For transplantation into the kidney capsule, the kidney was exteriorized through a dorsal-horizontal incision. A syringe with a 29G needle with a flattened tip was introduced into the kidney at a site away from the transplanted region. The kidney was penetrated, the tip of the needle positioned just beneath the renal capsule, and then the cell suspension of hiPSCs (1×10^5^ cells/10 µl of serum-free medium) was then injected underneath the kidney capsule. The mice were examined daily, and tumors were measured with calipers. The teratoma samples were resected and fixed with 4% (v/v) phosphate-buffered formalin, and paraffin-embedded sections were stained using with hematoxylin and eosin (H&E staining) according to standard procedures.

### lmmunocytochemical Staining

Cells were fixed in 4% PFA and washed with PBS. The fixed samples were permeabilized with 0.1% Triton X-100, blocked with 10% normal goat serum, and then reacted with the following Abs overnight at 4°C: anti-α-fetoprotein (AFP) Ab (SantaCruz, Santa Cruz, CA), anti-Cytokeratin19 Ab (clone A53-B/A2, Santa Cruz), anti-Desmin Ab (Thermo, Waltham, MA), anti-α-smooth muscle actin (SMA) Ab (clone 1A4, Dako, Carpinteria, CA), anti-glial fibrillary acidic protein (GFAP) Ab (Sigma, St. Louis, MO), anti-βIII-tubulin Ab (clone TuJ1, Babco, Richmond, CA), anti-OCT3/4 Ab (Chemicon), and anti-NANOG Ab (Reprocell, Tokyo, Japan). After several washes, the samples were incubated with the appropriate secondary Abs (AlexaFluor®-488-conjugated goat anti-mouse IgG Ab, AlexaFluor®-568-conjugated goat anti-rabbit IgG Ab, Molecular Probes, Invitrogen), and TO-PRO-3® iodide (1 mM, Molecular Probes, Invitrogen) for 1 hour at RT.

The samples were examined using a confocal laser scanning microscope (LSM510, Carl Zeiss, Hallbergmoos, Germany). All stainings were performed with matched isotype controls.

### Statistical Analysis

Statistical differences in the number of hESC-like colonies were determined by the Kruskal-Wallis test, and differences in gene expression levels obtained by qRT-PCR were determined by unpaired Student’s t-tests or ANOVA with post-hoc comparisons. Details are given in the figure legends.

## Results

### Generation of hiPSCs on PCM-DM

First, we examined the feasibility of generating hiPSCs on PCM-DM. After transducing DMCs with the four Yamanaka factors (OSKM) by retroviruses, the DMCs were seeded on PCM-DM and cultured with MEF-CM (Fig. 1A). After 30 days of induction, the hESC-like colonies were picked up, replated on PCM-DM (passage 1), the culture medium was changed to StemPro medium (passage 2), and the cells were further propagated (Fig. 1A). Among the colonies, we selected two representative hiPSC-PCMDM clones (clone 1, iPS-DMC72-PCMDM01; clone 2, iPS-DMC72-PCMDM02), which had an hESC-like morphology, characterized by large nuclei, scant cytoplasm, and flat-dense colonies, and analyzed their pluripotent stem cell properties in detail at early (about passage 10) and late (after passage 20) culture times.

### Characterization of hiPSC-PCMDM at Early and Late Culture Times

**Figure 2:**
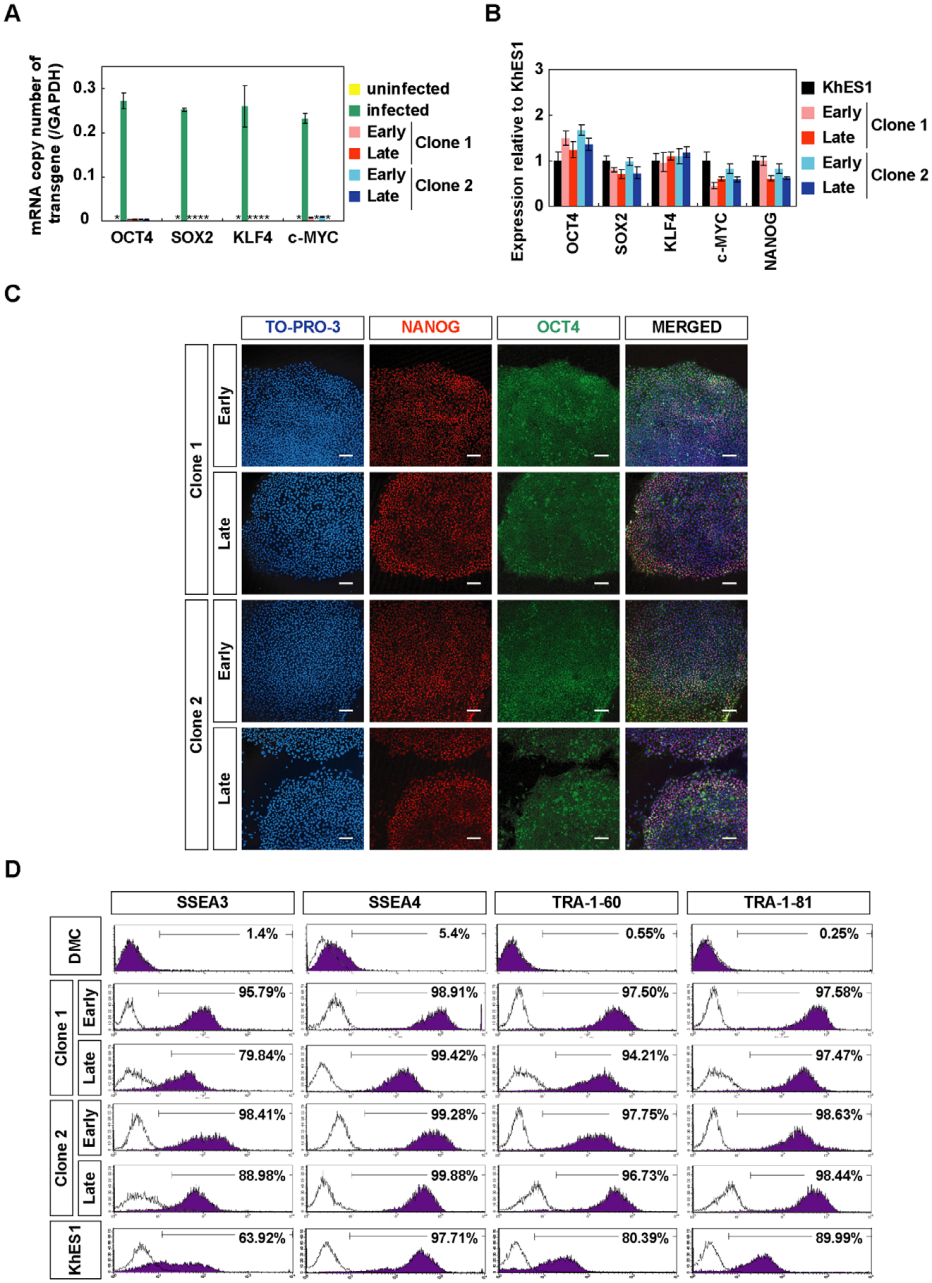
In vitro characterization of hiPSCs generated on PCM-DM. A) Quantitative RT-PCR analysis for the mRNA copy number of four transgenes (OCT4, SOX2, KLF4, c-MYC). All the transgenes were silenced in two hiPSC-PCMDM clones. Data are presented as the mean ± SD. Clone 1, iPS-DMC72-PCMDM01; Clone 2, iPS-DMC72-PCMDM02; Early, passage 8; Late, passage 30; *: not detected. B) Quantitative RT-PCR analysis for hESC marker gene (OCT4, SOX2, KLF4, c-MYC, NANOG) expression at early (passage 8) and late (passage 30) culture times compared with hESCs (clone KhES1). Data are presented as the mean ± SD. C) Immunocytochemistry for NANOG (red) and OCT4 (green) expression in hiPSC-PCMDM clones. Clone 1, Early (passage 10), Late (passage 22); Clone 2, Early (passage 11), Late (passage 25). Scale bar = 200 µm. D) Flow cytometry analysis for hESC-specific surface antigens (SSEA-3, SSEA-4, TRA-1-60, and TRA-1-81) at early (passage 10) and late (passage 30) culture times in comparison with parental DMCs and hESCs (KhES1). Clone 1, iPS-DMC72-PCMDM01; Clone2, iPS-DMC72-PCMDM02.

The two established hiPSC-PCMDM clones showed good proliferation activity, and retained their hESC-like morphology and ALP activity at the early and late passages (Fig. 1B, C). Expression analysis of the four transgenes OSKM by qRT-PCR showed that the mRNA copy number of each was well suppressed at the early and late culture times, in contrast to day 3 after infection (Fig. 2A). To examine the endogenous expression of OSKM and NANOG, we quantified their transcript level by qRT-PCR and compared it to the level in hESCs (KhES1) (Fig. 2B). Although the SOX2, c-MYC, and NANOG expression tended to be lower in the hiPSC-PCMDM clones than in KhES1, all these undifferentiated marker genes were expressed within an acceptable range of variation in the two hiPSC-PCMDM clones (Fig. 2B).

To examine the protein levels of hESC-marker molecules, we analyzed the two established clones by immunocytochemistry and FCM. Immunocytochemistry showed that the two clones stably expressed both OCT4 and NANOG in their nuclei at the early and late culture times (Fig. 2C). FCM analysis revealed that the clones highly expressed hESC-specific surface antigens (SSEA-3, SSEA-4, TRA-1-60, and TRA-1-81), at levels that were similar to KhES1 and stable between the early and late culture times (Fig. 2D). These findings indicated that the two established hiPSC-PCMDM clones were almost identical to hESCs in their undifferentiated state, and that their cellular properties were highly stable over long periods in culture.

### In vitro Differentiation

**Figure 3:**
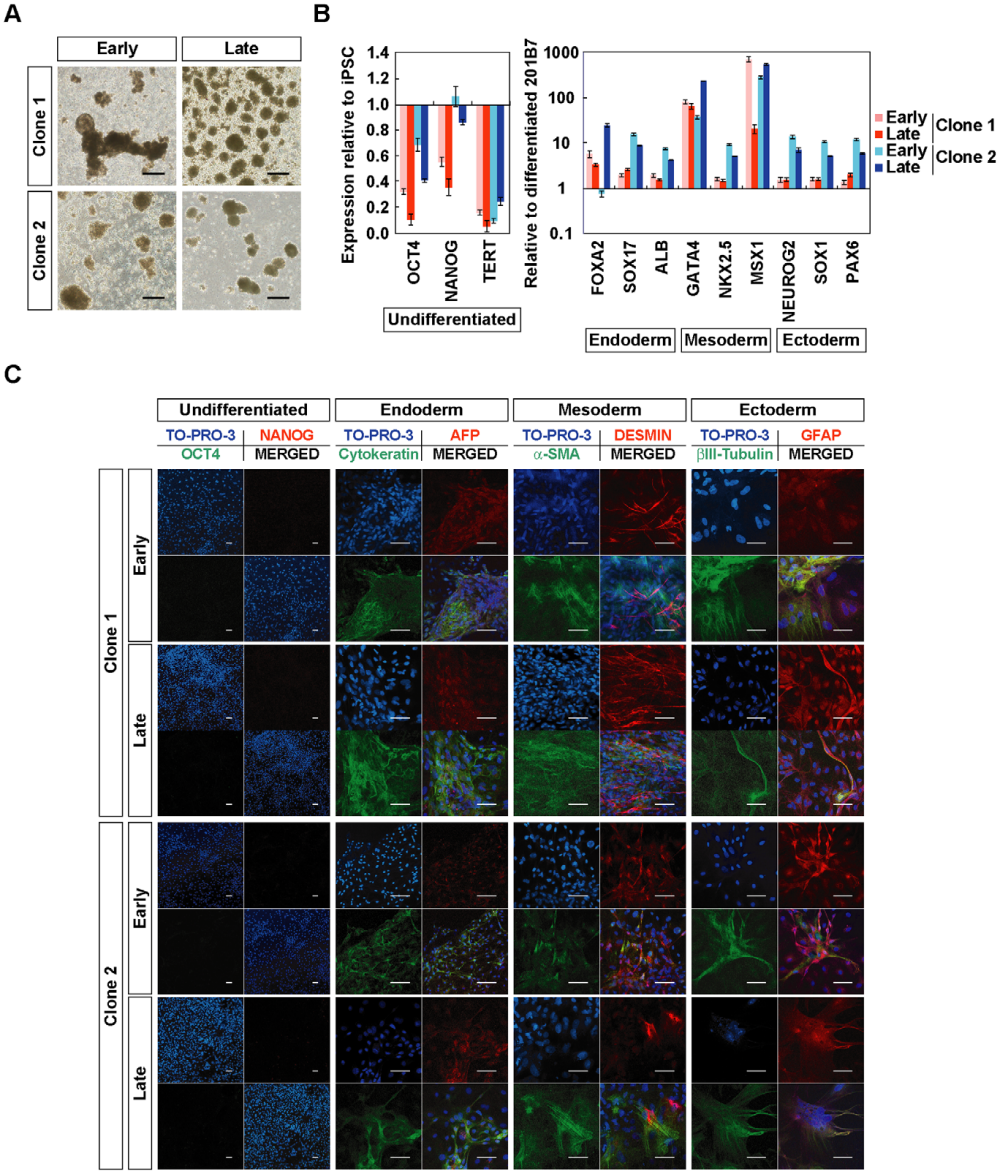
In vitro differentiation of hiPSCs generated on PCM-DM at early and late passages. A) Embryoid bodies (EBs) on day 8, derived from hiPSCs generated on PCM-DM at early (Clone 1, passage 11; Clone 2, passage 12) and late (Clone 1, passage 22; Clone 2, passage 21) culture times. Scale bar = 200 µm. B) Quantitative RT-PCR analysis. Left: Expression levels of undifferentiated genes in EBs relative to the hiPSCs before differentiation. Right: Expression levels of lineage-specific genes in EBs relative to differentiated 201B7. Data are presented as the mean ± SD. C) Immunocytochemical staining of differentiated cells by culturing EBs on gelatin-coated chamber slides with DMEM containing 10% FBS for 1 week. Clone 1, iPS-DMC72-PCMDM01; Clone 2, iPS-DMC72-PCMDM02. Scale bar = 50 µm.

The in vitro pluripotency of the two hiPSC-PCMDM clones was examined by EB formation assay. After 8 days of culture in petri dishes without bFGF, both clones formed EBs, whether the cells were taken from the early or late passages (Fig. 3A). Quantitative RT-PCR analysis showed that the OCT4 and TERT expressions were generally lower after EB formation than before, in both clones (Fig. 3B). NANOG expression was also suppressed in clone 1 (early and late passages); however, it was not markedly suppressed in clone 2 at either passage (Fig. 3B).

The expressions of lineage-specific (endoderm, mesoderm, and ectoderm) marker genes after the 8 days of EB formation were examined by qRT-PCR analysis and compared with those of differentiated hiPSCs (201B7) [1]. The expression levels of nine marker genes in EBs were equivalent to or higher than those in differentiated 201B7 regardless of the clone or culture time (Fig. 3B). To confirm the progression of differentiation, we cultured the EBs on gelatin-coated chamber slides for 8 days, and then with 10% FBS-containing medium for an additional week. After this 15-day total in vitro differentiation, the samples were examined by immunocytochemistry. At this point, the cells in the EBs had differentiated into various adherent cells, most of which expressed little OCT4 or NANOG (Fig. 3C). Some of these adherent cells expressed endoderm (AFP or cytokeratin), mesoderm (desmin or α-SMA), or ectoderm (GFAP or βIII-tubulin) marker proteins (Fig. 3C). These results indicated that the two established hiPSC-PCMDM clones had in vitro pluripotency, which was stably maintained in feeder-free culture on PCM-DM for over 20 passages.

### Karyotyping, Genotyping, and Promoter Methylation Analyses

**Figure 4:**
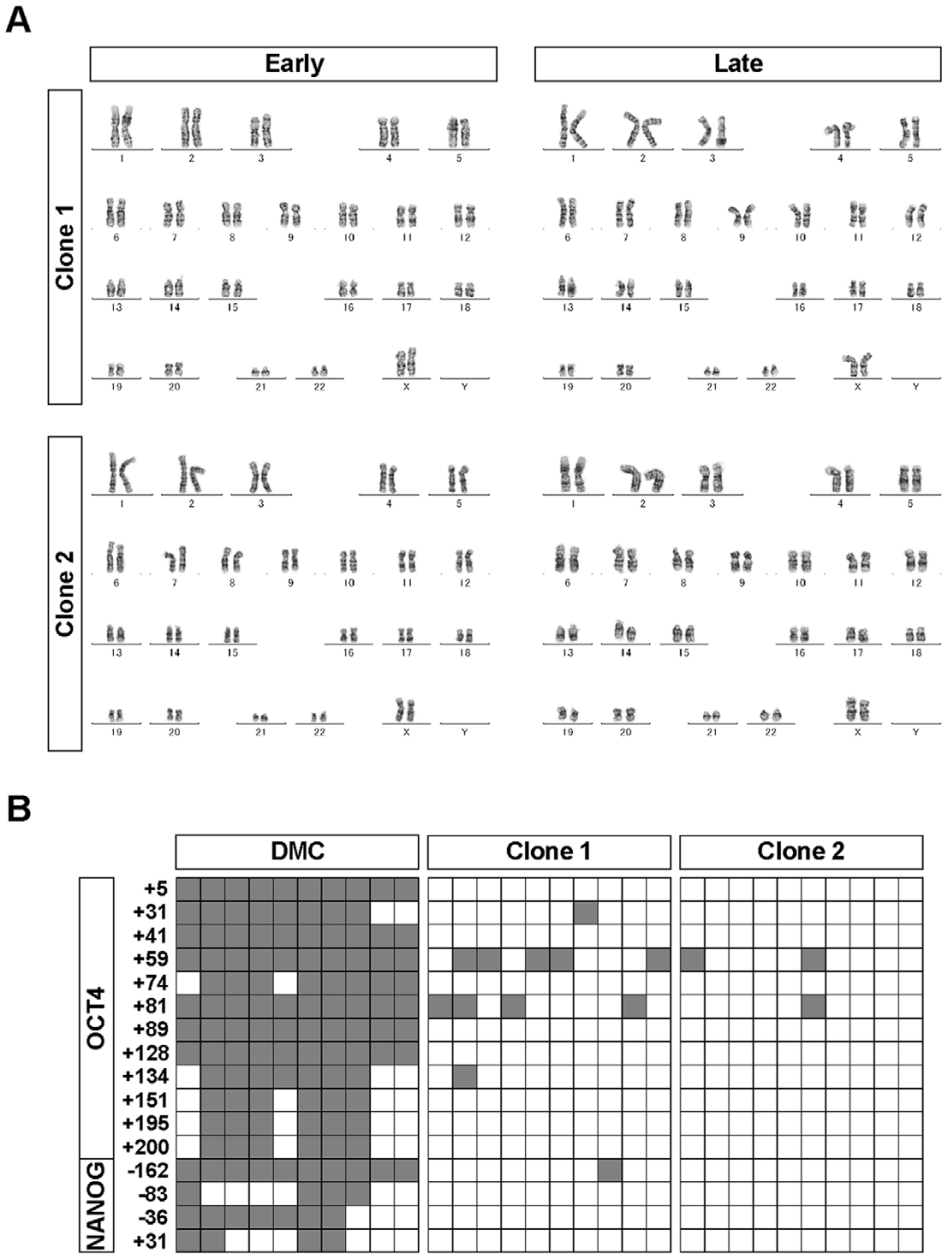
Karyotype and promoter methylation analyses of hiPSCs generated on PCM-DM. A) G-band staining of the two hiPSC-PCMDM clones showing a normal female karyotype (46, XX) in both clones at early (Clone 1, passage 12; Clone 2, passage 12) and late (Clone 1, passage 23; Clone 2, passage 24) passages. B) Methylation states of the OCT4 and NANOG promoter of the two hiPSC-PCMDM clones (passage 29) using bisulphate sequencing. Numbers indicate the position from the transcription start site. Open squares indicate unmethylated and filled squares indicate methylated CpG dinucleotides. Clone 1, iPS-DMC72-PCMDM01; Clone 2, iPS-DMC72-PCMDM02.

Karyotyping by G-band staining showed that both hiPSC-PCMDM clones had a normal female karyotype (46, XX) at the early and late culture times (Fig. 4A). Analysis of the methylation states of the OCT4 and NANOG promoter regions revealed that most of the CpG sites were unmethylated in the two hiPSC-PCMDM clones; in contrast, those of the parental DMCs were highly methylated (Fig. 4B). Genotyping by STR-PCR showed that the STRs completely matched between the parental DMCs and the two established clones (Supplemental Table S3). These findings showed that the two hiPSC-PCMDM clones were derived from the parental DMCs, and that their epigenetic states appeared to be reprogrammed efficiently. Furthermore, their karyotypes remained normal in the feeder-free culture on PCM-DM for over 20 passages.

### Teratoma Formation from hiPSC-PCMDM Clones at Early and Late Passages

**Figure 5:**
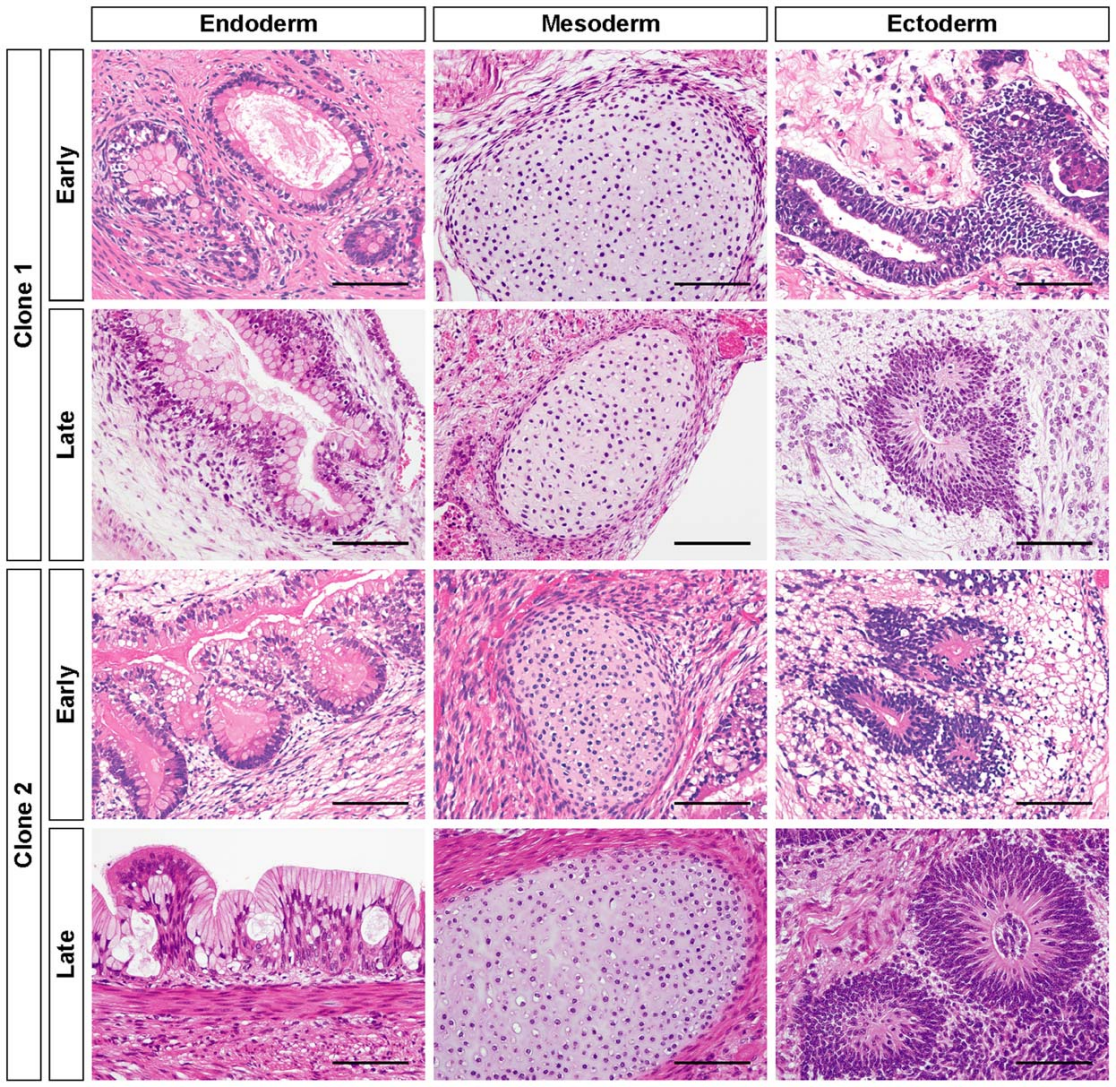
In vivo differentiation of hiPSCs generated on PCM-DM at early and late passages. hiPSC-PCMDM-induced teratomas were excised from mice and processed for H&E staining. Clone 1, iPS-DMC72-PCMDM01; Clone 2, iPS-DMC72-PCMDM02. Early, passage 12; Late, passage 23; Scale bar = 100 µm.

The pluripotency of the two clones was also evaluated by *in vivo* teratoma formation assay. Both clones, whether cultured for short or longer times, equally formed tumor masses in NOG mice after several months, and these masses contained various histological components of the three germ layers. The tumors partly showed neural rosette-like structures (ectoderm), cartilage-like structures (mesoderm), or gut-like epithelium (endoderm) (Fig. 5). These findings indicated that both hiPSC-PCMDM clones could form teratomas showing in vivo pluripotency, and that their in vivo pluripotency was fully preserved after feeder-free culture on PCM-DM for over 20 passages. Collectively, these findings confirmed that the two established hiPSC-PCMDM clones met the criteria for hiPSCs, and thus, the feeder-free generation and long-term maintenance of hiPSCs on PCM-DM was successful.

### Global Gene Expression of hiPSC-PCMDM

**Figure 6:**
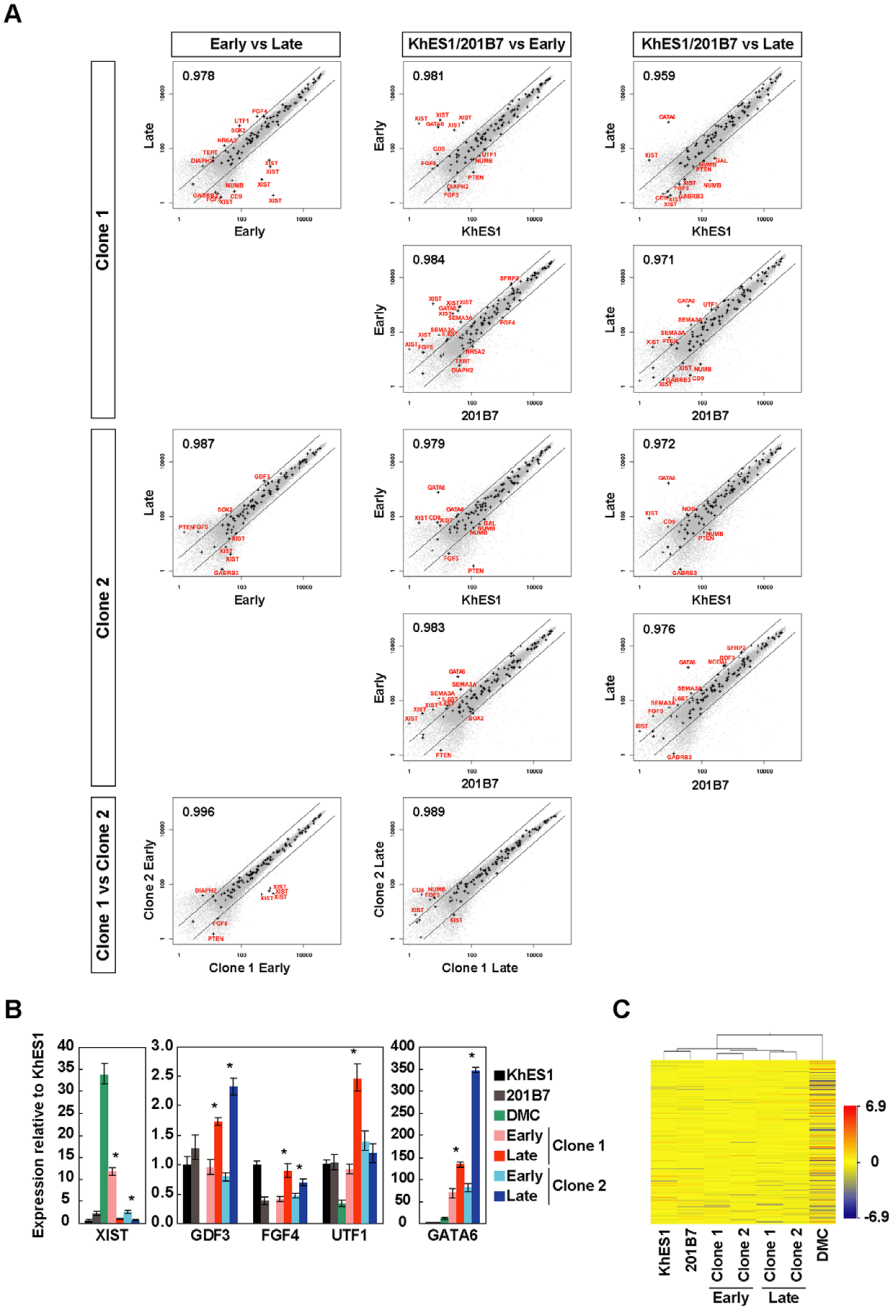
Global gene expression analysis. A) Global gene expression of the hiPSC-PCMDM clones, KhES1, and 201B7 by microarray analysis. Scatter plots and Pearson’s coefficient are shown. The diagonal lines indicate 3-fold changes in gene expression levels. Plus (“+”) symbols indicate stem-cell marker genes suggested by the International Stem Cell Initiative [31], and such genes outside the 3-fold change lines are shown in red text. Early, passage 8; Late, passage 30. Clone 1, iPS-DMC72-PCMDM01; Clone 2, iPS-DMC72-PCMDM02. B) Quantitative RT-PCR analysis of the XIST, GDF3, FGF4, UTF1, and GATA6 genes. Data are presented as the mean ± SD. Early, passage 8; Late, passage 30. Statistical differences between early and late passage group are determined by unpaired Student’s t-test (*, P<0.01). C) Hierarchical cluster analysis between parental DMCs, KhES1 cells, 201B7, and the two hiPSC-PCMDM clones at early (passage 8) and late (passage 30) passages.

To further characterize the hiPSC-PCMDM clones at the molecular level, we examined their genome-wide gene-expression profiles and compared them with those of hESCs (KhES1) and hiPSCs (201B7). Microarray analysis showed that the global gene expression profiles of the hiPSC-PCMDM clones at early and late passages were very similar, and that the differences between the two clones were very small (Fig. 6A). However, some gene expressions changed over time (Fig. 6A, B). When we focused on the expressions of a stem cell marker gene set suggested by the International Stem Cell Initiative [31], some genes (clone 1: fibroblast growth factor 4 [FGF4] and undifferentiated embryonic cell transcription factor 1 [UTF1]; clone 2: growth differentiation factor-3 [GDF3]) were more highly expressed at the late passage (Fig. 6A, Supplemental Table S4). On the other hand, the expression of the X (inactive)-specific transcript (non-protein coding) gene (XIST) in hiPSC-PCMDM clone 1 was markedly suppressed at late passages, to the level seen in hiPSC-PCMDM clone 2 (Fig. 6A, Supplemental Table S4). Moreover, comparison of the gene expression of the hiPSC-PCMDM clones with that of KhES1 or 201B7 showed that GATA-binding factor 6 (GATA6) was more highly expressed in both clones than in KhES1 or 201B7 from the early passage (Fig. 6A); these results were confirmed by qRT-PCR analysis (Fig. 6B).

Finally, cluster analysis showed that the two hiPSC-PCMDM clones were clearly separated from the parental DMCs and the clusters of KhES1 and 201B7 (Fig. 6C). Interestingly, the clones from the early passage and late passage also clustered separately. These results indicate that the gene-expression patterns of the hiPSC-PCMDM clones were similar to those of KhES1 and 201B7 but not to those of the parental DMCs. Moreover, the differences in the established hiPSC-PCMDM clones between the early and late passages were relatively small, but they trended similarly as the number of passages increased.

### Expression of GATA6 in hiPSC-PCMDM

**Figure 7:**
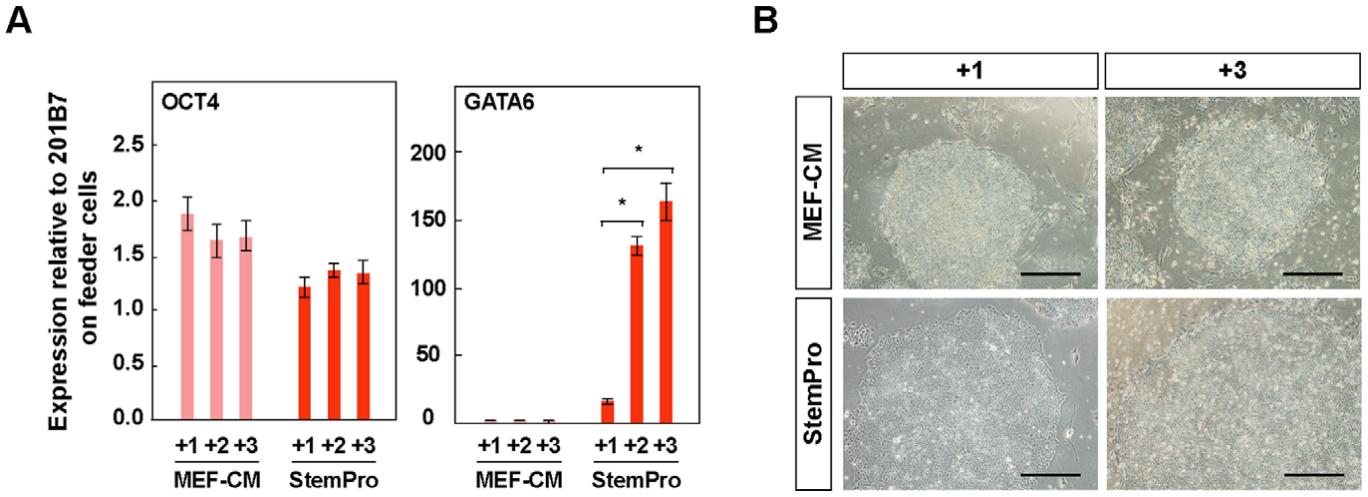
Increased GATA6 expression in 201B7 on PCM-DM with StemPro medium. A) Quantitative RT-PCR analysis of OCT4 and GATA6 for 201B7 cultured on PCM-DM with MEF-CM or StemPro medium. “+number” indicates the passage number after reseeding on PCM-DM. Statistical significances are determined by Scheffe’s test after two-way ANOVA. Results of comparisons among groups of medium within each passage are shown (*, P<0.01). B) Morphology of 201B7 cultured on PCM-DM with MEF-CM or StemPro medium. Scale bar = 500 µm.

The factors affecting GATA6 gene expression were also examined. An established iPSC clone (201B7), which was routinely propagated on SNL feeder cells and in hESC medium, was reseeded on PCM-DM using MEF-CM or StemPro medium, and the gene expressions of OCT4 and GATA6 were examined for 3 passages (Fig. 7A, B). OCT4 expression was almost the same under both culture conditions (Fig. 7A). In contrast, GATA6 expression was significantly up-regulated in the cells cultured in StemPro medium and was not expressed in those cultured in MEF-CM (Fig. 7A). These findings indicated that the GATA6 expression might be induced by the StemPro medium and not by PCM-DM.

### Reprogramming Efficiency Using PCM-DM Versus Other Substrates

We also established hiPSC clones (iPS-DMC71-PCMDM and iPS-DMC92-PCMDM) by the same method, using PCM-DM with MEF-CM. These two clones had a clear hESC-like morphology and ALP activity (Supplemental Figure S1A). Expression analysis by qRT-PCR showed that the mRNA copy number of the four transgenes OSKM was fully suppressed (Supplemental Figure S1B) and that the endogenous expression levels of OSKM and NANOG were within the acceptable range of variation, compared with the level in hESCs (Supplemental Figure S1C). Immunocytochemistry showed that the two clones stably expressed both OCT4 and NANOG in their nuclei (Supplemental Figure S1D). These findings showed that the feeder-free generation and culture of hiPSCs using PCM-DM is feasible and reproducible.

**Figure 8:**
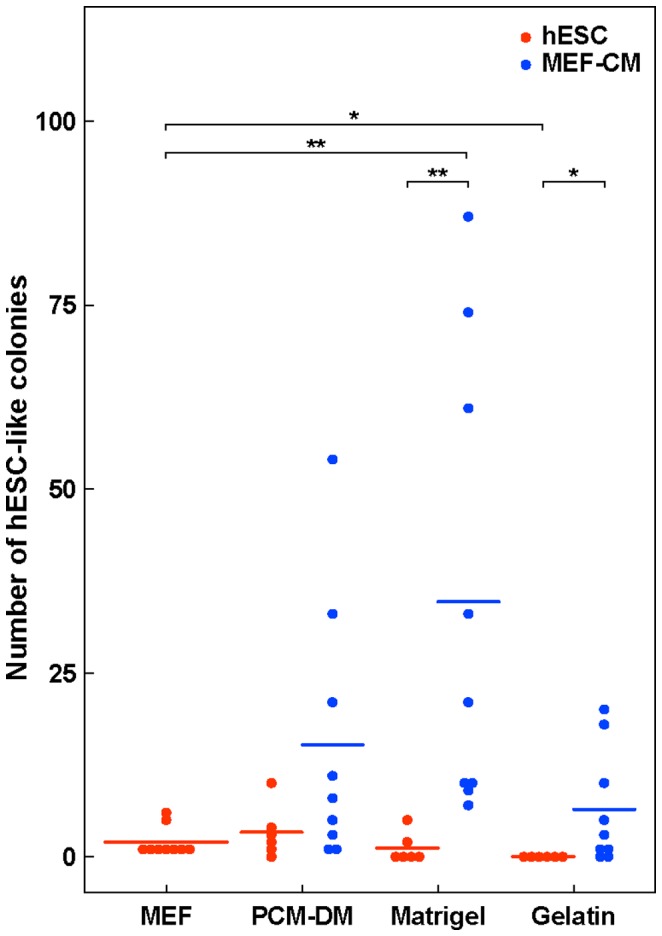
Comparison of reprogramming efficiency. Number of hESC-like colonies derived from DMCs under seven different conditions: MEF with hESC medium, PCM-DM with NC-hESC medium, Matrigel with NC-hESC medium, gelatin with NC-hESC medium, PCM-DM with MEF-CM, Matrigel with MEF-CM, and gelatin with MEF-CM. Horizontal bars indicate the mean for each method. Statistical differences were determined by the Kruskal-Wallis test (*, p<0.05, **, P<0.01).

To examine the applicability of iPSC generation using PCM-DM further, we compared the reprogramming efficiency for six different lines of DMCs under seven different culture conditions as follows: plated on MEFs with NC-hESC medium (control), on PCM-DM with MEF-CM, on Matrigel with MEF-CM, on gelatin with MEF-CM, on PCM-DM with NC-hESC medium, on Matrigel with NC-hESC medium, and on gelatin with NC-hESC medium. In the cultures with MEF-CM, the most hESC-like colonies appeared on Matrigel, with a statistically significant efficiency; fewer, but still significant, numbers of hESC-like colonies were also obtained using PCM-DM or gelatin (Fig. 8, Supplemental Table S5). On the other hand, in the cultures using NC-hESC medium, we obtained hESC-like colonies at lower levels, with similar efficiencies, on PCM-DM and Matrigel (Fig. 8, Supplemental Table S5). In contrast, no colonies with clear hESC-like characteristics were obtained from DMCs cultured on gelatin with NC-hESC medium (Fig. 8, Supplemental Table S5). These findings suggest that PCM-DM could be used to generate hiPSCs with a reprogramming efficiency that was almost as good or the same as that of other substrates used with MEF-CM or NC-hESC medium.

### Cellular Properties of hiPSCs Generated on PCM-DM in Non-conditioned Medium

**Figure 9:**
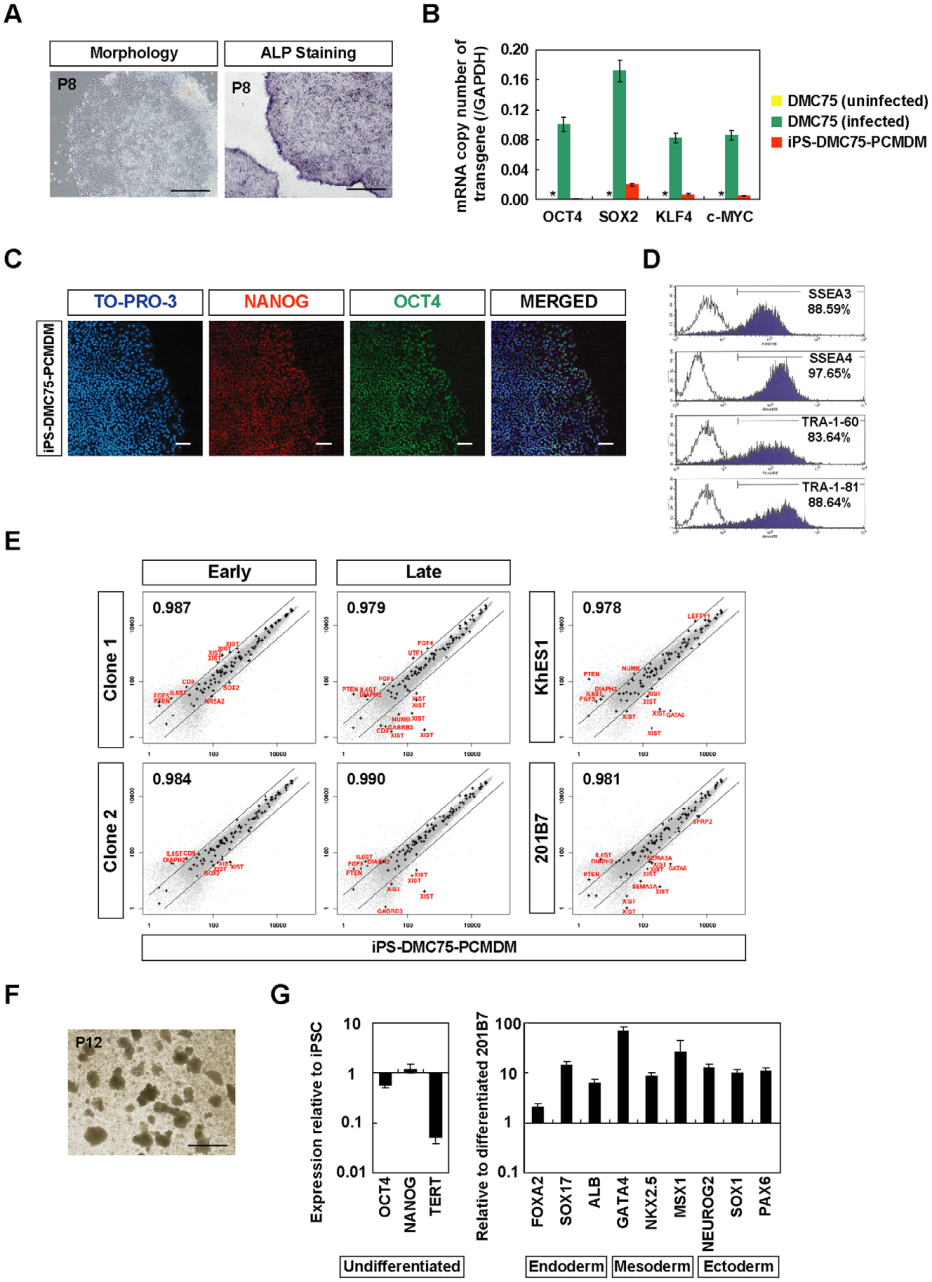
Generation of hiPSCs from DMCs with non-conditioned hESC medium on PCM-DM. A) Morphology and Alkaline phosphatase (ALP) staining of iPS-DMC75-PCMDM. P, passage number. Scale bar = 500 µm. B). Quantitative RT-PCR analysis for the mRNA copy number of four transgenes (OCT4, SOX2, KLF4, c-MYC). All the transgenes were silenced in iPS-DMC75-PCMDM. Data are presented as the mean ± SD. *: not detected. C) Immunocytochemistry for NANOG (red) and OCT4 (green) expression in iPS-DMC75-PCMDM. Scale bar = 200 µm. D) Flow cytometry analysis for hESC-specific surface antigens (SSEA-3, SSEA-4, TRA-1-60, and TRA-1-81) at passage 11. E) Global gene expression analysis of iPS-DMC75-PCMDM (passage 12), early and late passages of clones 1 and 2, KhES1, and 201B7. Scatter plots and Pearson’s coefficient are shown. The diagonal lines indicate 3-fold changes in gene expression levels. Plus (“+”) symbols indicate stem-cell marker genes suggested by the International Stem Cell Initiative [31], and such genes outside the 3-fold change lines are shown in red text. Clone 1, iPS-DMC72-PCMDM01; Clone 2, iPS-DMC72-PCMDM02. F) Embryoid bodies (EBs) on day 8 derived from iPS-DMC75-PCMDM (passage 12). Scale bar = 500 µm. G) Quantitative RT-PCR analysis. Left, Expression levels of genes for the undifferentiated state in EBs relative to hiPSCs before differentiation. Right, Expression levels of lineage-specific genes in EBs relative to differentiated 201B7. Data are presented as the mean ± SD.

Finally, to examine the applicability of hiPSCs generated on PCM-DM without MEF-CM, we examined the detailed cellular properties of hiPSCs generated on PCM-DM in NC-hESC medium. One representative clone (iPS-DMC75-PCMDM), which was initially reprogramed on PCM-DM with NC-hESC medium and further propagated on PCM-DM with StemPro medium after 2 passages, retained its hESC-like morphology and had ALP activity (Fig. 9A). Expression analysis of the four transgenes OSKM by qRT-PCR showed that the mRNA copy number of each was suppressed (Fig. 9B). Immunocytochemistry showed that this clone stably expressed both OCT4 and NANOG in its nuclei (Fig. 9C). FCM analysis revealed that this clone highly expressed hESC-specific surface antigens (SSEA-3, SSEA-4, TRA-1-60, and TRA-1-81) (Fig. 9D), and microarray analysis showed that its global gene expression patterns were similar to the early and late passages of clones 1 and 2, as well as to KhES1 and 201B7 (Fig. 9E). Moreover, iPS-DMC75-PCMDM expressed GATA6 at a higher level than KhES1 or 201B7, and at about the same level as clones 1 and 2 (Fig. 9E). This clone formed EBs (Fig. 9F) and differentiated into the three germ layers, as assessed on the gene expression level (Fig. 9G), although the expression of the undifferentiated marker NANOG persisted for 8 days after the start of differentiation. These findings show that it is feasible to generate hiPSCs on PCM-DM without MEF-CM.

## Discussion

### Feeder-cell-free Generation of hiPSCs on PCM-DM

It is easier to control the quality of feeder-cell-free cultures than of those that use human-derived primary cells, which makes them more attractive for clinical applications. In general, the characteristics of human-derived primary cells (e.g., dermal fibroblasts) that are used for xenobiotic-free culture methods vary widely from line to line, and can only be passaged a small number of times. Moreover, their feeder-cell activity for hiPSCs/hESCs can vary from batch to batch [27]. To overcome these variabilities, various xenobiotic- and feeder-cell-free methods have been developed. Of these, Matrigel [6]–[9] has been widely used as a standard control. In addition to Matrigel, several other materials, including laminin-511 [14], [15], fibronectin [10]–[13], vitronectin [32]–[35], collagen I [36], and E-cadherin [37] exhibit maintenance activity for hiPSCs/hESCs, and fibronectin was reported to support hiPSC generation [10]. More complex and mixed materials like human-serum matrix [38], human fibroblast extracellular matrix [39], [40], and autologous extracellular matrix (from H9 EB derived-cells) [41] are also reported to be useful for culturing hiPSCs/hESCs.

In this study, we examined PCM-DM as a feeder-cell-free method for generating hiPSCs, because it is a human-derived material with the ability to maintain hiPSCs/hESCs equivalent to that of Matrigel [23]. Human iPSCs generated on PCM-DM showed clear hESC-compatible phenotypes in their undifferentiated state, and they differentiated into three germ layers in vitro and in vivo. These hESC-like properties of hiPSC-PCMDM were fully maintained for at least 20 passages, and detailed analysis using microarrays showed that the global gene expression profiles of the hiPSC-PCMDM clones were also quite stable over 20 passages (Fig. 6). Moreover, we succeeded in establishing additional hiPSC clones (iPS-DMC71-PCMDM and iPS-DMC92-PCMDM) by the same method, using PCM-DM with MEF-CM, and these clones also had hESC-like properties (Supplemental Fig. S1). These findings indicated that the generation and long-term maintenance of hiPSCs on PCM-DM, with no exposure to feeder cells, is feasible and reproducible, and that the cell biological properties of the hiPSCs are well retained on PCM-DM.

Although we have not yet identified the molecular components of PCM-DM responsible for supporting the self-renewal and pluripotency of the hiPSCs/hESCs because of its complexity, PCM-DM is reported to include fibronectin and collagen IV but very little laminin [23]. Therefore, the properties of PCM-DM may be different from laminin-based materials such as Matrigel, which is mainly composed of laminin-111, or human recombinant laminin-511 [14], [15]. PCM-DM and Matrigel, which is also complex, show higher activity for maintaining hESCs than their individual components such as fibronectin [11], [23]; of these, PCM-DM as a human-derived material may be more useful for medical applications.

### Gene Expression Properties of hiPSC-PCMDM

The cellular properties of the hiPSC-PCMDM clones were almost identical to those of hiPSCs generated and maintained on feeder cells; however, some interesting differences were found. We found higher expression levels of FGF4 and UTF1 in clone 1 and of GDF3 in clone 2 at the late passage. FGF4 and UTF1 are target genes of OCT4 and SOX2 [42], [43]. In our study, the OCT4 and SOX2 expressions were almost unchanged between the early and late passages in both clones (Fig. 2B), and FGF4 and UTF1 showed only small changes when compared with KhES1 (Fig. 6B). We think that these differences in FGF4 and UTF1 expression seen in clone 1 were acceptable dispersion that is unlikely to significantly affect the cellular properties of clone 1. GDF3 has been identified in Activin-treated embryonic carcinoma cells, and it contributes to the maintenance of hESCs [44]–[46]. In this study, starting at passage 2, we used the StemPro medium, which contains Activin (10 ng/ml) and might gradually induce GDF3 over time in culture, although MEF-CM also contains Activin [47].

In contrast, XIST was more highly expressed in clone 1 at the early passage than in KhES1 or 201B7. The expression level of XIST, which is involved in the inactivation of chromosome X in female cells, appeared to decrease gradually toward the level in KhES1 and 201B7 with long-term culture. A high expression level of XIST in DMCs is reasonable, because DMCs are female-derived cells and XIST helps elicit the dosage compensation for chromosome X [48]. Given that KhES1 and 201B7 are also female-derived cells and showed low XIST expression, and some female hiPSCs [49] and hESCs [50]–[53] are reported to show decreasing expression of XIST during culture, our hiPSC clones may resemble good hiPSCs in this respect. That is, the decreased XIST expression in female hiPSCs and hESCs may indicate a global epigenetic status that is specific for pluripotent stem cells [49].

In addition to XIST, interestingly, GATA6 was more highly expressed in our two established clones than in KhES1 or 201B7 throughout the long culture period. GATA6 is considered to be a marker for primitive or definitive endoderm in early embryogenesis [54], [55]. Although the hiPSC-PCMDM clones showed full pluripotency as a mass both in vitro and in vivo, the expression of GATA6 in hiPSC-PCMDM might indicate that some cells spontaneously differentiated into extra-embryonic tissues or definitive endoderm. On the other hand, our examinations using 201B7 showed that the GATA6 expression in hiPSCs cultured on PCM-DM was completely repressed by MEF-CM, but not by StemPro medium. This finding indicated that the GATA6 expression may be induced by the StemPro medium, not by the PCM-DM itself.

StemPro medium is a defined culture medium developed for hiPSCs/hESCs; it contains Activin A, FGF2, ErbB-2 ligand HRG-1 beta, and insulin-like growth factor ligand LR3-IGF1 [56]. We previously confirmed that the combination of StemPro medium and PCM-DM was useful for maintaining hiPSCs/hESCs [23]. Activin can induce the differentiation of definitive endoderm from hESCs [57]. Although Activin is also present in MEF-CM, McLean et al reported that hESCs cultured with MEF-CM and Matrigel could differentiate into definitive endoderm only when the phosphatidylinositol 3-kinases (PI3K) signaling pathway was blocked [55]. In addition to Activin, KSR and insulin are present in MEF-CM, and these factors act as agonists of the PI3K-signaling pathway and thus could suppress definitive endoderm differentiation induced by Activin [55]. Furthermore, unknown factors contained in the MEF-CM might positively and strongly suppress the activity of Activin and the progression of differentiation into definitive endoderm.

### Feasibility of Feeder-cell-free hiPSC Generation on PCM-DM with Non-conditioned Medium

In this study, we used MEF-CM in the initial phase of hiPSC generation, i.e., until the second passage, and StemPro medium thereafter. In the preliminary phase of this study, we tried using StemPro medium from the beginning of hiPSC generation, but we failed to generate hiPSCs, most likely owing to the limited proliferation of DMCs in StemPro medium. On the other hand, our analysis of the reprogramming efficiency of six different DMC lines under seven different culture conditions also showed that feeder-cell-free hiPSC generation on PCM-DM is feasible with both MEF-CM and NC-hESC medium, with comparable efficiency as on other substrates, and that clones generated and propagated without MEF-CM could stably maintain the cellular properties of hiPSCs. These findings suggest that MEF-CM is dispensable for generating hiPSCs on PCM-DM, and that various types of culture medium may be effective for generating hiPSCs on PCM-DM.

Although the most hESC-like colonies appeared on Matrigel with MEF-CM, both Matrigel and MEF-CM contain xenobiotic components, and thus may not be suitable for future clinical applications. Previous reports showed that hiPSCs can be generated from fibroblasts on gelatin-coated plates with NC-hESC medium [27] and that DMCs, like fibroblasts, have some maintenance activity for hESC/hiPSCs [23]. However, no hiPSC colonies were generated from DMCs on gelatin with NC-hESC medium (Fig. 8, Supplemental Table S5). This finding may indicate that the properties of DMCs acting as an auto-feeder on gelatin may be insufficient to generate hiPSCs in non-conditioned medium, and that feeder-cell-free generation on gelatin with non-conditioned medium might require modifications to generate different cell types. Our comparison of feeder-cell-free culture systems showed that culturing on PCM-DM is convenient, stable, and compatible with both MEF-CM and non-conditioned medium.

Taken together, our present results suggest that the combination of PCM-DM and StemPro medium might be slightly worse at maintaining hESCs/hiPSCs in an undifferentiated condition than the combination of PCM-DM and MEF-CM. Nevertheless, the pluripotency of the hiPSC-PCMDM was retained for over 20 passages, and the feeder-cell-free culture of hiPSCs using PCM-DM is practical and useful. Further improvements to the culture medium should increase the stability of the feeder-free-generated and cultured hiPSCs, and should be a topic of future studies.

### PCM-DM may be a Useful Human-derived Matrix for Regenerative Medicine

Extra-embryonic tissues such as the umbilical cord and placenta have been suggested as attractive sources for human cells to be used in regenerative medicine. In this study, we used DMCs isolated from the decidua membrane, which is the maternal potion of the placenta [26]. DMCs exhibit a typical fibroblast-like morphology and have a high proliferative potential for over 30 population doublings, which is better than that of BM-MSCs [26]. They strongly express the mesenchymal cell marker vimentin, but not cytokeratin 19 or HLA-G, and FCM analysis showed that their expression pattern of cell-surface antigens closely resembles that of BM-MSCs [26]. In vitro, DMCs show good differentiation into chondrocytes and moderate differentiation into adipocytes, but little evidence of osteogenesis, compared with BM-MSCs [26]. These findings indicate that DMCs are mesenchymal cells of purely maternal origin, and that they are unique cells with MSC-like properties but differ from BM-MSCs. The greater proliferative ability of DMCs means that their cultivation might require less maintenance, and their derivation from the maternal portion of human fetal adnexal tissues, which are otherwise discarded, would resolve many ethical concerns associated with the use of embryonic cells. Moreover, the high success rate of DMC isolation from tissues stored more than 24 hours indicates that it might be feasible to develop a system for collecting or banking fetal adnexal tissues from multiple or even remote hospitals [26]. These properties of DMCs identify them as easily accessible human source for clinical uses. Our findings indicate that the generation of hiPSCs on PCM-DM, which has several advantages for clinical use, provides an opportunity to establish hiPSCs for clinical applications.

### Conclusion

We generated hiPSCs that were stably maintained with respect to their self-renewal, pluripotency, and genome integrity over long-term culture on PCM-DM. Our findings indicate that PCM-DM can maintain and support the generation of hiPSCs. We suggest that PCM-DM is a practical and easily accessible, human-derived substrate that can be used, not just for the stable maintenance of hiPSCs, but also for their generation.

## Supporting Information

Figure S1 **Generation of hiPSCs from DMCs on PCM-DM.** A) Morphology and Alkaline phosphatase (ALP) staining of iPS-DMC71-PCMDM and iPS-DMC92-PCMDM. P, passage number. Scale bar = 500 µm. B) Quantitative RT-PCR analysis for the mRNA copy number of four transgenes (OCT4, SOX2, KLF4, c-MYC). All the transgenes were silenced in the two hiPSC-PCMDM clones. Data are presented as the mean ± SD. *: not detected. B) Quantitative RT-PCR analysis for hESC marker gene (OCT4, SOX2, KLF4, c-MYC, NANOG) expression compared with hESCs (clone KhES1). Data are presented as the mean ± SD. C) Immunocytochemistry for NANOG (red) and OCT4 (green) expression in two hiPSC-PCMDM clones. Scale bar = 200 µm.
(TIF)

Table S1 **Primers for quantitative RT-PCR.**
(DOC)

Table S2 **Primers for detecting the OCT4 and NANOG promoter.**
(DOC)

Table S3 **Results of short tandem repeat PCR (STR-PCR).**
(DOC)

Table S4 **Probe Set in Affymetrix Human Genome U133 Plus 2.0 Array for characterization of undifferentiated stem cells.**
(DOC)

Table S5 **Reprogramming efficiencies.**
(DOC)

## References

[1] Takahashi K, Tanabe K, Ohnuki M, Narita M, Ichisaka T, et al (2007) Induction of pluripotent stem cells from adult human fibroblasts by defined factors. Cell 131: 861–872. 18035408 10.1016/j.cell.2007.11.019

[2] Yu J, Vodyanik MA, Smuga-Otto K, Antosiewicz-Bourget J, Frane JL, et al (2007) Induced pluripotent stem cell lines derived from human somatic cells. Science 318: 1917–1920. 18029452 10.1126/science.1151526

[3] Thomson JA, Itskovitz-Eldor J, Shapiro SS, Waknitz MA, Swiergiel JJ, et al (1998) Embryonic stem cell lines derived from human blastocysts. Science 282: 1145–1147. 9804556 10.1126/science.282.5391.1145

[4] Reubinoff BE, Pera MF, Fong CY, Trounson A, Bongso A (2000) Embryonic stem cell lines from human blastocysts: somatic differentiation in vitro. Nat Biotechnol 18: 399–404. 10748519 10.1038/74447

[5] Pan C, Hicks A, Guan X, Chen H, Bishop CE (2010) SNL fibroblast feeder layers support derivation and maintenance of human induced pluripotent stem cells. J Genet Genomics 37: 241–248. 20439100 10.1016/S1673-8527(09)60042-4 PMC3768137

[6] Xu C, Inokuma MS, Denham J, Golds K, Kundu P, et al (2001) Feeder-free growth of undifferentiated human embryonic stem cells. Nat Biotechnol 19: 971–974. 11581665 10.1038/nbt1001-971

[7] Rosler ES, Fisk GJ, Ares X, Irving J, Miura T, et al (2004) Long-term culture of human embryonic stem cells in feeder-free conditions. Dev Dyn 229: 259–274. 14745951 10.1002/dvdy.10430

[8] Sun N, Panetta NJ, Gupta DM, Wilson KD, Lee A, et al (2009) Feeder-free derivation of induced pluripotent stem cells from adult human adipose stem cells. Proc Natl Acad Sci U S A 106: 15720–15725. 19805220 10.1073/pnas.0908450106 PMC2739869

[9] Navarro-Alvarez N, Soto-Gutierrez A, Yuasa T, Yamatsuji T, Shirakawa Y, et al (2008) Long-term culture of Japanese human embryonic stem cells in feeder-free conditions. Cell Transplant 17: 27–33. 18468232

[10] Hayashi Y, Chan T, Warashina M, Fukuda M, Ariizumi T, et al (2010) Reduction of N-glycolylneuraminic acid in human induced pluripotent stem cells generated or cultured under feeder- and serum-free defined conditions. PLoS One 5: e14099. 21124894 10.1371/journal.pone.0014099 PMC2990711

[11] Lu J, Hou R, Booth CJ, Yang SH, Snyder M (2006) Defined culture conditions of human embryonic stem cells. Proc Natl Acad Sci U S A 103: 5688–5693. 16595624 10.1073/pnas.0601383103 PMC1458634

[12] Amit M, Shariki C, Margulets V, Itskovitz-Eldor J (2004) Feeder layer- and serum-free culture of human embryonic stem cells. Biol Reprod 70: 837–845. 14627547 10.1095/biolreprod.103.021147

[13] Kitajima H, Niwa H (2010) Clonal expansion of human pluripotent stem cells on gelatin-coated surface. Biochem Biophys Res Commun 396: 933–938. 20460107 10.1016/j.bbrc.2010.05.026

[14] Rodin S, Domogatskaya A, Strom S, Hansson EM, Chien KR, et al (2010) Long-term self-renewal of human pluripotent stem cells on human recombinant laminin-511. Nat Biotechnol 28: 611–615. 20512123 10.1038/nbt.1620

[15] Miyazaki T, Futaki S, Hasegawa K, Kawasaki M, Sanzen N, et al (2008) Recombinant human laminin isoforms can support the undifferentiated growth of human embryonic stem cells. Biochem Biophys Res Commun 375: 27–32. 18675790 10.1016/j.bbrc.2008.07.111

[16] Martin MJ, Muotri A, Gage F, Varki A (2005) Human embryonic stem cells express an immunogenic nonhuman sialic acid. Nat Med 11: 228–232. 15685172 10.1038/nm1181

[17] Klimanskaya I, Rosenthal N, Lanza R (2008) Derive and conquer: sourcing and differentiating stem cells for therapeutic applications. Nat Rev Drug Discov 7: 131–142. 18079756 10.1038/nrd2403

[18] Hovatta O, Mikkola M, Gertow K, Stromberg AM, Inzunza J, et al (2003) A culture system using human foreskin fibroblasts as feeder cells allows production of human embryonic stem cells. Hum Reprod 18: 1404–1409. 12832363 10.1093/humrep/deg290

[19] Cheng L, Hammond H, Ye Z, Zhan X, Dravid G (2003) Human adult marrow cells support prolonged expansion of human embryonic stem cells in culture. Stem Cells 21: 131–142. 12634409 10.1634/stemcells.21-2-131

[20] Richards M, Tan S, Fong CY, Biswas A, Chan WK, et al (2003) Comparative evaluation of various human feeders for prolonged undifferentiated growth of human embryonic stem cells. Stem Cells 21: 546–556. 12968109 10.1634/stemcells.21-5-546

[21] Amit M, Margulets V, Segev H, Shariki K, Laevsky I, et al (2003) Human feeder layers for human embryonic stem cells. Biol Reprod 68: 2150–2156. 12606388 10.1095/biolreprod.102.012583

[22] Anchan RM, Quaas P, Gerami-Naini B, Bartake H, Griffin A, et al (2011) Amniocytes can serve a dual function as a source of iPS cells and feeder layers. Hum Mol Genet 20: 962–974. 21156717 10.1093/hmg/ddq542 PMC3033187

[23] Nagase T, Ueno M, Matsumura M, Muguruma K, Ohgushi M, et al (2009) Pericellular matrix of decidua-derived mesenchymal cells: a potent human-derived substrate for the maintenance culture of human ES cells. Dev Dyn 238: 1118–1130. 19384957 10.1002/dvdy.21944

[24] Suemori H, Yasuchika K, Hasegawa K, Fujioka T, Tsuneyoshi N, et al (2006) Efficient establishment of human embryonic stem cell lines and long-term maintenance with stable karyotype by enzymatic bulk passage. Biochem Biophys Res Commun 345: 926–932. 16707099 10.1016/j.bbrc.2006.04.135

[25] Amit M, Carpenter MK, Inokuma MS, Chiu CP, Harris CP, et al (2000) Clonally derived human embryonic stem cell lines maintain pluripotency and proliferative potential for prolonged periods of culture. Dev Biol 227: 271–278. 11071754 10.1006/dbio.2000.9912

[26] Kanematsu D, Shofuda T, Yamamoto A, Ban C, Ueda T, et al (2011) Isolation and cellular properties of mesenchymal cells derived from the decidua of human term placenta. Differentiation 82: 77–88. 21684674 10.1016/j.diff.2011.05.010

[27] Takahashi K, Narita M, Yokura M, Ichisaka T, Yamanaka S (2009) Human induced pluripotent stem cells on autologous feeders. PLoS One 4: e8067. 19956543 10.1371/journal.pone.0008067 PMC2780725

[28] Livak KJ, Schmittgen TD (2001) Analysis of relative gene expression data using real-time quantitative PCR and the 2(-Delta Delta C(T)) Method. Methods 25: 402–408. 11846609 10.1006/meth.2001.1262

[29] Edgar R, Domrachev M, Lash AE (2002) Gene Expression Omnibus: NCBI gene expression and hybridization array data repository. Nucleic Acids Res 30: 207–210. 11752295 10.1093/nar/30.1.207 PMC99122

[30] Ito M, Hiramatsu H, Kobayashi K, Suzue K, Kawahata M, et al (2002) NOD/SCID/gamma(c)(null) mouse: an excellent recipient mouse model for engraftment of human cells. Blood 100: 3175–3182. 12384415 10.1182/blood-2001-12-0207

[31] Adewumi O, Aflatoonian B, Ahrlund-Richter L, Amit M, Andrews PW, et al (2007) Characterization of human embryonic stem cell lines by the International Stem Cell Initiative. Nat Biotechnol 25: 803–816. 17572666 10.1038/nbt1318

[32] Yap LY, Li J, Phang IY, Ong LT, Ow JZ, et al (2011) Defining a threshold surface density of vitronectin for the stable expansion of human embryonic stem cells. Tissue Eng Part C Methods 17: 193–207. 20726687 10.1089/ten.TEC.2010.0328

[33] Braam SR, Zeinstra L, Litjens S, Ward-van Oostwaard D, van den Brink S, et al (2008) Recombinant vitronectin is a functionally defined substrate that supports human embryonic stem cell self-renewal via alphavbeta5 integrin. Stem Cells 26: 2257–2265. 18599809 10.1634/stemcells.2008-0291

[34] Prowse AB, Doran MR, Cooper-White JJ, Chong F, Munro TP, et al (2010) Long term culture of human embryonic stem cells on recombinant vitronectin in ascorbate free media. Biomaterials 31: 8281–8288. 20674971 10.1016/j.biomaterials.2010.07.037

[35] Chen G, Gulbranson DR, Hou Z, Bolin JM, Ruotti V, et al (2011) Chemically defined conditions for human iPSC derivation and culture. Nat Methods 8: 424–429. 21478862 10.1038/nmeth.1593 PMC3084903

[36] Furue MK, Na J, Jackson JP, Okamoto T, Jones M, et al (2008) Heparin promotes the growth of human embryonic stem cells in a defined serum-free medium. Proc Natl Acad Sci U S A 105: 13409–13414. 18725626 10.1073/pnas.0806136105 PMC2522264

[37] Nagaoka M, Si-Tayeb K, Akaike T, Duncan SA (2010) Culture of human pluripotent stem cells using completely defined conditions on a recombinant E-cadherin substratum. BMC Dev Biol 10: 60. 20525219 10.1186/1471-213X-10-60 PMC2896937

[38] Stojkovic P, Lako M, Przyborski S, Stewart R, Armstrong L, et al (2005) Human-serum matrix supports undifferentiated growth of human embryonic stem cells. Stem Cells 23: 895–902. 15888688 10.1634/stemcells.2004-0326

[39] Escobedo-Lucea C, Stojkovic M (2010) Growth of human embryonic stem cells using derivates of human fibroblasts. Methods Mol Biol 584: 55–69. 19907971 10.1007/978-1-60761-369-5_3

[40] Meng G, Liu S, Li X, Krawetz R, Rancourt DE (2010) Extracellular matrix isolated from foreskin fibroblasts supports long-term xeno-free human embryonic stem cell culture. Stem Cells Dev 19: 547–556. 19883201 10.1089/scd.2009.0303

[41] Fu X, Toh WS, Liu H, Lu K, Li M, et al.. (2011) Establishment of Clinically Compliant Human Embryonic Stem Cells in an Autologous Feeder-Free System. Tissue Eng Part C Methods 927–937. 10.1089/ten.TEC.2010.0735 21561302

[42] Nishimoto M, Fukushima A, Okuda A, Muramatsu M (1999) The gene for the embryonic stem cell coactivator UTF1 carries a regulatory element which selectively interacts with a complex composed of Oct-3/4 and Sox-2. Mol Cell Biol 19: 5453–5465. 10409735 10.1128/mcb.19.8.5453 PMC84387

[43] Yuan H, Corbi N, Basilico C, Dailey L (1995) Developmental-specific activity of the FGF-4 enhancer requires the synergistic action of Sox2 and Oct-3. Genes Dev 9: 2635–2645. 7590241 10.1101/gad.9.21.2635

[44] Levine AJ, Brivanlou AH (2006) GDF3, a BMP inhibitor, regulates cell fate in stem cells and early embryos. Development 133: 209–216. 16339188 10.1242/dev.02192

[45] Levine AJ, Brivanlou AH (2006) GDF3 at the crossroads of TGF-beta signaling. Cell Cycle 5: 1069–1073. 16721050 10.4161/cc.5.10.2771

[46] Caricasole AA, van Schaik RH, Zeinstra LM, Wierikx CD, van Gurp RJ, et al (1998) Human growth-differentiation factor 3 (hGDF3): developmental regulation in human teratocarcinoma cell lines and expression in primary testicular germ cell tumours. Oncogene 16: 95–103. 9467948 10.1038/sj.onc.1201515

[47] Beattie GM, Lopez AD, Bucay N, Hinton A, Firpo MT, et al (2005) Activin A maintains pluripotency of human embryonic stem cells in the absence of feeder layers. Stem Cells 23: 489–495. 15790770 10.1634/stemcells.2004-0279

[48] Shofuda T, Kanematsu D, Fukusumi H, Yamamoto A, Bamba Y, et al.. (2013) Human Decidua-Derived Mesenchymal Cells are a Promising Source for the Generation and Banking of Human Induced Pluripotent Stem Cells. Cell Med: in press. 10.3727/215517912X658918 PMC4733846 26858858

[49] Tchieu J, Kuoy E, Chin MH, Trinh H, Patterson M, et al (2010) Female human iPSCs retain an inactive X chromosome. Cell Stem Cell 7: 329–342. 20727844 10.1016/j.stem.2010.06.024 PMC2935700

[50] Silva SS, Rowntree RK, Mekhoubad S, Lee JT (2008) X-chromosome inactivation and epigenetic fluidity in human embryonic stem cells. Proc Natl Acad Sci U S A 105: 4820–4825. 18339803 10.1073/pnas.0712136105 PMC2290799

[51] Lengner CJ, Gimelbrant AA, Erwin JA, Cheng AW, Guenther MG, et al (2010) Derivation of pre-X inactivation human embryonic stem cells under physiological oxygen concentrations. Cell 141: 872–883. 20471072 10.1016/j.cell.2010.04.010

[52] Shen Y, Matsuno Y, Fouse SD, Rao N, Root S, et al (2008) X-inactivation in female human embryonic stem cells is in a nonrandom pattern and prone to epigenetic alterations. Proc Natl Acad Sci U S A 105: 4709–4714. 18339804 10.1073/pnas.0712018105 PMC2290804

[53] Hall LL, Byron M, Butler J, Becker KA, Nelson A, et al (2008) X-inactivation reveals epigenetic anomalies in most hESC but identifies sublines that initiate as expected. J Cell Physiol 216: 445–452. 18340642 10.1002/jcp.21411 PMC3057623

[54] Vallier L, Touboul T, Chng Z, Brimpari M, Hannan N, et al (2009) Early cell fate decisions of human embryonic stem cells and mouse epiblast stem cells are controlled by the same signalling pathways. PLoS One 4: e6082. 19564924 10.1371/journal.pone.0006082 PMC2700259

[55] McLean AB, D’Amour KA, Jones KL, Krishnamoorthy M, Kulik MJ, et al (2007) Activin a efficiently specifies definitive endoderm from human embryonic stem cells only when phosphatidylinositol 3-kinase signaling is suppressed. Stem Cells 25: 29–38. 17204604 10.1634/stemcells.2006-0219

[56] Wang L, Schulz TC, Sherrer ES, Dauphin DS, Shin S, et al (2007) Self-renewal of human embryonic stem cells requires insulin-like growth factor-1 receptor and ERBB2 receptor signaling. Blood 110: 4111–4119. 17761519 10.1182/blood-2007-03-082586 PMC2190616

[57] D’Amour KA, Agulnick AD, Eliazer S, Kelly OG, Kroon E, et al (2005) Efficient differentiation of human embryonic stem cells to definitive endoderm. Nat Biotechnol 23: 1534–1541. 16258519 10.1038/nbt1163

