# Supplementary figures and images for: Feeder-Free Generation and Long-Term Culture of Human Induced Pluripotent Stem Cells Using Pericellular Matrix of Decidua Derived Mesenchymal Cells
Source: PLoS One. 2013 Jan 31;8(1):e55226. doi: 10.1371/journal.pone.0055226 (PMC3561375; doi:10.1371/journal.pone.0055226)

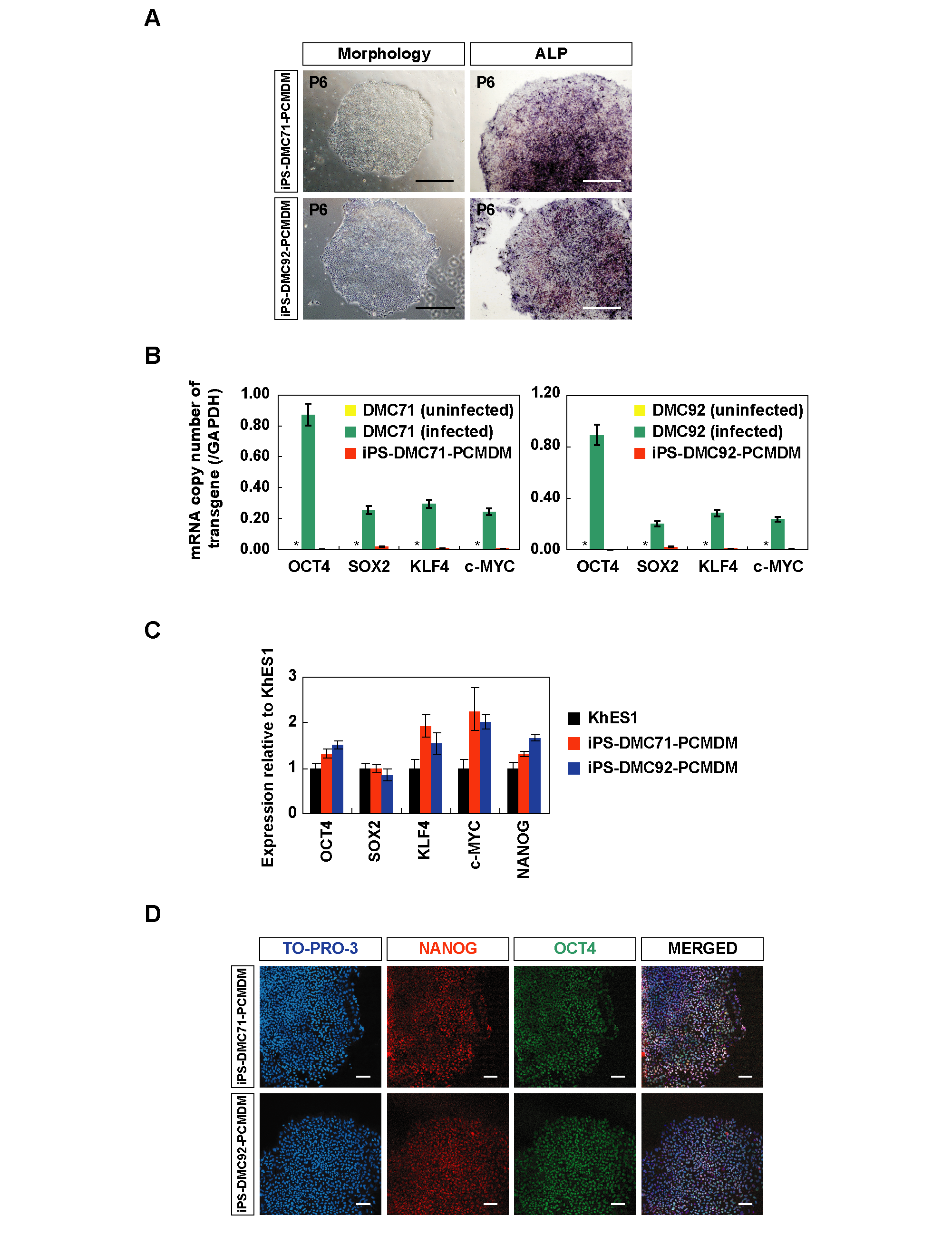

Supplement: Figure S1 — Generation of hiPSCs from DMCs on PCM-DM. A) Morphology and Alkaline phosphatase (ALP) staining of iPS-DMC71-PCMDM and iPS-DMC92-PCMDM. P, passage number. Scale bar = 500 µm. B) Quantitative RT-PCR analysis for the mRNA copy number of four transgenes (OCT4, SOX2, KLF4, c-MYC). All the transgenes were silenced in the two hiPSC-PCMDM clones. Data are presented as the mean ± SD. *: not detected. B) Quantitative RT-PCR analysis for hESC marker gene (OCT4, SOX2, KLF4, c-MYC, NANOG) expression compared with hESCs (clone KhES1). Data are presented as the mean ± SD. C) Immunocytochemistry for NANOG (red) and OCT4 (green) expression in two hiPSC-PCMDM clones. Scale bar = 200 µm. (TIF) [file pone.0055226.s001.tif]
