# Supplementary material for: Feeder-Free Generation and Long-Term Culture of Human Induced Pluripotent Stem Cells Using Pericellular Matrix of Decidua Derived Mesenchymal Cells
Source: PLoS One. 2013 Jan 31;8(1):e55226. doi: 10.1371/journal.pone.0055226 (PMC3561375; doi:10.1371/journal.pone.0055226)
Supplement: Table S1 — Primers for quantitative RT-PCR. (DOC) [file pone.0055226.s002.doc]

**Table S1:Primers for quantitative RT-PCR**

| Gene Name | Accession | Forward Primer (5' to 3') | Reverse Primer (5' to 3') |
| --- | --- | --- | --- |
| Transgene detection | | | |
| OCT4 | - | CCCCAGGGCCCCATTTTGGTACC | TTATCGTCGACCACTGTGCTGCTG |
| SOX2 | - | GGCACCCCTGGCATGGCTCTTGGCTC |
| KLF4 | - | ACGATCGTGGCCCCGGAAAAGGACC |
| c-MYC | - | AACAACCGAAAATGCACCAGCCCCAG |
| Undifferentiated marker | | | |
| OCT4 | NM_002701 | GACAGGGGGAGGGGAGGAGCTAGG | CTTCCCTCCAACCAGTTGCCCCAAAC |
| SOX2 | NM_003106 | GGGAAATGGGAGGGGTGCAAAAGAGG | TTGCGTGAGTGTGGATGGGATTGGTG |
| KLF4 | NM_004235 | ACGATCGTGGCCCCGGAAAAGGACC | TGATTGTAGTGCTTTCTGGCTGGGCTCC |
| c-MYC | NM_002467 | GCGTCCTGGGAAGGGAGATCCGGAGC | TTGAGGGGCATCGTCGCGGGAGGCTG |
| NANOG | NM_024865 | GCAGAAGGCCTCAGCACCTA | GGTTCCCAGTCGGGTTCAC |
| TERT | NM_198253 | CTCCATCCTGAAAGCCAAGAA | CGAGTCAGCTTGAGCAGGAA |
| Endoderm marker | | | |
| FOXA2 | NM_021784 | TGCTGGTCGTTTGTTGTGG | CATGTTGCTCACGGAGGAGTAG |
| SOX17 | NM_022454 | CCCATAGTTGGATTGTCAAAACC | CACACCCAGGACAACATTTCTTT |
| ALB | NM_000477 | GTTGCATGAGAAAACGCCAGTA | AGCATGGTCGCCTGTTCAC |
| Mesoderm marker | | | |
| GATA4 | NM_002052 | CTCTTCAGGCAGTGAGAGCC | GGTCCGTGCAGGAATTTGAGG |
| NKX2.5 | NM_004387 | CCCCTGGATTTTGCATTCAC | CGTGCGCAAGAACAAACG |
| MSX1 | NM_002448 | CAGAAGATGCGCTCGTCAAA | CGGCTTACGGTTCGTCTTG |
| Ectoderm marker | | | |
| NEUROG2 | NM_024019 | ATGCCTATTGTCCTGTCCTTCTCT | TGACTTCTAACCTGCCCCTCTAAC |
| SOX1 | NM_005986 | AGCAGTTGTTTCTGGAAGAGTCTGT | AGGCCCTTATCCCGGACTAA |
| PAX6 | NM_000280 | ACCTGGCTAGCGAAAAGCAA | CCCGTTCAACATCCTTAGTTTATCA |
| Differential expression | | | |
| XIST | NR_001564 | TGGCAGGGAGTGCCAGCTCCA | GACCAAGGTGCATGGCTGCGGT |
| GDF3 | NM_020634 | CGGGAATGTACTTCGCTTTCTC | TTTCTTTGATGGCAGACAGGTT |
| FGF4 | NM_002007 | GCTGGGCATCAAGCGGCTGC | CTCCAGCAGGCTGTCGCGGG |
| UTF1 | NM_003577 | GCTGCAGACCCTGGGGCACC | GCCCACGGCCAGGGACACTG |
| GATA6 | NM_005257 | TCTACAGCAAGATGAACGGCCTCA | TCTGCGCCATAAGGTGGTAGTTGT |
| Control | | | |
| GAPDH | NM_002046 | CCACTTTGTCAAGCTCATTTCCT | TCTCTTCCTCTTGTGCTCTTGCT |
