# Supplementary material for: Feeder-Free Generation and Long-Term Culture of Human Induced Pluripotent Stem Cells Using Pericellular Matrix of Decidua Derived Mesenchymal Cells
Source: PLoS One. 2013 Jan 31;8(1):e55226. doi: 10.1371/journal.pone.0055226 (PMC3561375; doi:10.1371/journal.pone.0055226)
Supplement: Table S2 — Primers for detecting the OCT4 and NANOG promoter. (DOC) [file pone.0055226.s003.doc]

**Table S2: Primers for detecting the OCT4 and NANOG promoter**

| Gene Name | Accession | Forward Primer (5' to 3') | Reverse Primer (5' to 3') |
| --- | --- | --- | --- |
| OCT4 | NC_000006 | GTTAAGGTTAGTGGGTGGGATT | AACATAAAAAAATCCCCCACA |
| NANOG | NC_000012 | GTTGGGTTTGTTTTTAGGTTTT | CATAAAACAACCAACTCAATCC |
