# Supplementary material for: Feeder-Free Generation and Long-Term Culture of Human Induced Pluripotent Stem Cells Using Pericellular Matrix of Decidua Derived Mesenchymal Cells
Source: PLoS One. 2013 Jan 31;8(1):e55226. doi: 10.1371/journal.pone.0055226 (PMC3561375; doi:10.1371/journal.pone.0055226)
Supplement: Table S3 — Results of short tandem repeat PCR (STR-PCR). (DOC) [file pone.0055226.s004.doc]

**Table S3: Results of short tandem repeat PCR (STR-PCR)**

| Locus/Lot | DMC | Clone 1 | Clone 2 |
| --- | --- | --- | --- |
| CSF1PO | 10,13 | 10,13 | 10,13 |
| D13S317 | 12 | 12 | 12 |
| D16S539 | 9,10 | 9,10 | 9,10 |
| D18S51 | 11,15 | 11,15 | 11,15 |
| D21S11 | 29,31 | 29,31 | 29,31 |
| D3S1358 | 15 | 15 | 15 |
| D5S818 | 10,11 | 10,11 | 10,11 |
| D7S820 | 13 | 13 | 13 |
| D8S1179 | 13,16 | 13,16 | 13,16 |
| FGA | 19,21 | 19,21 | 19,21 |
| Penta_D | 9,13 | 9,13 | 9,13 |
| Penta_E | 11,18 | 11,18 | 11,18 |
| TH01 | 9,10 | 9,10 | 9,10 |
| TPOX | 11,12 | 11,12 | 11,12 |
| vWA | 17 | 17 | 17 |
| AMEL | X | X | X |
