# Supplementary material for: Feeder-Free Generation and Long-Term Culture of Human Induced Pluripotent Stem Cells Using Pericellular Matrix of Decidua Derived Mesenchymal Cells
Source: PLoS One. 2013 Jan 31;8(1):e55226. doi: 10.1371/journal.pone.0055226 (PMC3561375; doi:10.1371/journal.pone.0055226)
Supplement: Table S4 — Probe Set in Affymetrix Human Genome U133 Plus 2.0 Array for characterization of undifferentiated stem cells. (DOC) [file pone.0055226.s005.doc]

| **Table S4: Probe Set in Affymetrix Human Genome U133 Plus 2.0 Array for characterization of undifferentiated stem cells** | Fold Change versus KhES1 | Clone 2 | Late | -1.12 | 1.31 | -3.49 | 4.98 | -1.92 | -1.07 | 1.09 | -1.08 | 1.12 | -1.05 | 1.43 | -1.17 | 1.95 | 1.04 | -3.8 | 1.4 | -1.22 | 1.35 | -1.91 | 1.09 | -1.01 | 1.58 | 1.02 | 1.19 | 1.66 | -18.97 | -2.5 | -1.59 | 189.45 |
| --- | --- | --- | --- | --- | --- | --- | --- | --- | --- | --- | --- | --- | --- | --- | --- | --- | --- | --- | --- | --- | --- | --- | --- | --- | --- | --- | --- | --- | --- | --- | --- | --- |
|  |  |  | Early | 1.38 | 1.21 | -2.16 | 7.02 | -1.9 | -1.18 | 1.03 | -1.02 | 1.3 | 1.04 | 1.07 | -1.01 | 1.01 | -2.5 | 1.29 | -4.48 | -1.05 | -1.37 | -9.71 | 1.02 | 1.43 | 1.42 | -1.26 | -1.14 | 1.45 | 1.19 | -3.45 | -1.79 | 85.97 |
|  |  | Clone 1 | Late | -1.59 | 1.72 | -10.2 | -3.2 | -2.1 | 1.3 | -1 | 1.03 | -1.36 | 1.52 | 1.73 | 1.16 | 2.08 | 1.68 | 2.24 | -3.99 | -2.67 | 2.26 | -7.96 | 1.21 | -1.05 | 1.89 | 1.13 | 1.04 | 2.16 | -8.63 | -6.06 | -2.57 | 105.54 |
|  |  |  | Early | 1.4 | 1.3 | -3.41 | 7.51 | -2.1 | -1.4 | 1.13 | -1.11 | -4.94 | 1.09 | 1.28 | 1.29 | 1.14 | -2.67 | 1.84 | -6.51 | 3.13 | -1.1 | -1.61 | 5.66 | 1.45 | 1.37 | 1.18 | 1.1 | 2.33 | -1.37 | -2.92 | -1.25 | 70.06 |
|  | Fold Change versus DMC | Clone 2 | Late | 1.66 | 1.86 | 1.9 | 1.08 | -10.12 | 7.66 | 33.18 | 7.66 | 2.25 | 124.85 | 361.04 | 62.73 | 1104.03 | 17.43 | -1.11 | -9.95 | -73.92 | -4.42 | 18.32 | 2.87 | 834.29 | 22.42 | 161.58 | 547.59 | 53.41 | -1.39 | 27.19 | 759.61 | 8.13 |
|  |  |  | Early | 2.56 | 1.72 | 3.07 | 1.52 | -10.01 | 6.94 | 31.28 | 8.13 | 2.61 | 137.22 | 271.06 | 72.11 | 573.48 | 6.69 | 4.39 | -62.29 | -64.02 | -8.12 | 3.6 | 2.69 | 1211.78 | 20.17 | 126.34 | 401.44 | 46.51 | 16.31 | 19.71 | 677.08 | 3.69 |
|  |  | Clone 1 | Late | 1.17 | 2.45 | -1.54 | -14.79 | -11.07 | 10.68 | 30.29 | 8.54 | 1.48 | 199.79 | 438.64 | 84.8 | 1180.68 | 28.09 | 7.64 | -55.48 | -162.31 | -2.64 | 4.38 | 3.18 | 807.94 | 26.73 | 179.82 | 479.65 | 69.44 | 1.59 | 11.21 | 471.23 | 4.53 |
|  |  |  | Early | 2.6 | 1.85 | 1.94 | 1.63 | -11.1 | 5.86 | 34.44 | 7.47 | -2.46 | 142.57 | 323.49 | 94.16 | 647.61 | 6.26 | 6.28 | -90.5 | -19.42 | -6.52 | 21.69 | 14.88 | 1229.34 | 19.35 | 188.02 | 504.35 | 74.83 | 9.98 | 23.25 | 967.4 | 3.01 |
|  | Flag * | DMC | | P | P | A | A | P | P | P | P | M | P | A | A | A | A | A | P | P | P | A | A | A | A | A | A | A | A | A | A | P |
|  |  | Clone 2 | Late | P | P | A | A | P | P | P | P | A | P | P | P | P | P | A | A | A | P | A | A | P | P | P | P | P | A | P | P | P |
|  |  |  | Early | P | P | A | P | P | P | P | P | P | P | P | P | P | P | A | A | A | A | A | A | P | P | P | P | P | P | P | P | P |
|  |  | Clone 1 | Late | P | P | A | A | P | P | P | P | A | P | P | P | P | P | A | A | A | A | A | A | P | P | P | P | P | A | A | P | P |
|  |  |  | Early | P | P | A | A | P | P | P | P | A | P | P | P | P | P | A | A | A | A | A | A | P | P | P | P | P | A | P | P | P |
|  |  | KhES1 | | P | P | A | A | P | P | P | P | P | P | P | P | P | P | A | A | A | A | A | A | P | P | P | P | P | A | P | P | A |
|  | Probe Set ID | | | 219177_at | 201005_at | 233317_at | 233322_at | 218048_at | 202575_at | 205603_s_at | 205726_at | 217246_s_at | 220668_s_at | 204271_s_at | 204273_at | 206701_x_at | 206783_at | 1552982_a_at | 208378_x_at | 210310_s_at | 210311_at | 208500_x_at | 241609_at | 241612_at | 205850_s_at | 227690_at | 227830_at | 229724_at | 1569689_s_at | 207466_at | 214240_at | 210002_at |
|  | UniGene ID | | | Hs.718510 | Hs.114286 | Hs.114286 | Hs.114286 | Hs.534398 | Hs.405662 | Hs.226483 | Hs.226483 | Hs.226483 | Hs.643024 | Hs.82002 | Hs.82002 | Hs.82002 | Hs.1755 | Hs.1755 | Hs.37055 | Hs.37055 | Hs.37055 | Hs.546573 | Hs.546573 | Hs.546573 | Hs.302352 | Hs.302352 | Hs.302352 | Hs.302352 | Hs.302352 | Hs.278959 | Hs.278959 | Hs.514746 |
|  | Gene Title | | | brix domain containing 2 | CD9 molecule | CD9 molecule | CD9 molecule | COMM domain containing 3 | cellular retinoic acid binding protein 2 | diaphanous homolog 2 (Drosophila) | diaphanous homolog 2 (Drosophila) | diaphanous homolog 2 (Drosophila) | DNA (cytosine-5-)-methyltransferase 3 beta | endothelin receptor type B | endothelin receptor type B | endothelin receptor type B | fibroblast growth factor 4 | fibroblast growth factor 4 | fibroblast growth factor 5 | fibroblast growth factor 5 | fibroblast growth factor 5 | forkhead box D3 | Forkhead box D3 | forkhead box D3 | gamma-aminobutyric acid (GABA) A receptor, beta 3 | gamma-aminobutyric acid (GABA) A receptor, beta 3 | gamma-aminobutyric acid (GABA) A receptor, beta 3 | gamma-aminobutyric acid (GABA) A receptor, beta 3 | gamma-aminobutyric acid (GABA) A receptor, beta 3 | galanin prepropeptide | galanin prepropeptide | GATA binding protein 6 |
|  | Gene Symbol | | | BXDC2 | CD9 | CD9 | CD9 | COMMD3 | CRABP2 | DIAPH2 | DIAPH2 | DIAPH2 | DNMT3B | EDNRB | EDNRB | EDNRB | FGF4 | FGF4 | FGF5 | FGF5 | FGF5 | FOXD3 | FOXD3 | FOXD3 | GABRB3 | GABRB3 | GABRB3 | GABRB3 | GABRB3 | GAL | GAL | GATA6 |

| 2.74 | 3.06 | 2.61 | 2.26 | 2.02 | 1.64 | 1.93 | -1.06 | 1.21 | -1.37 | 1.69 | 2.13 | -1.03 | -1.01 | -3.15 | -6.16 | -1.02 | -1.33 | -1.9 | -1.22 | 1.18 | 1.35 | 1.2 | 1.06 | -1.22 | 1.7 | 1.16 | 3.54 | 1.52 | 1.33 | 1.3 | 4.91 | 2.52 | 6.05 | 1.79 | 1.29 | 1.38 | 1.06 |
| --- | --- | --- | --- | --- | --- | --- | --- | --- | --- | --- | --- | --- | --- | --- | --- | --- | --- | --- | --- | --- | --- | --- | --- | --- | --- | --- | --- | --- | --- | --- | --- | --- | --- | --- | --- | --- | --- |
| 3.08 | 9.94 | -1.33 | 1.31 | 1.41 | 1.01 | 1.65 | 1.2 | 1.06 | 1.42 | 1.59 | 1.88 | -1.13 | 1.17 | -1.96 | -4.95 | -2.1 | -1.37 | -1.4 | 1.34 | 1.6 | 1.54 | 1.04 | 1.1 | -1.2 | -1.1 | 1.11 | 2.83 | -1.37 | 1.1 | 1.2 | -3.35 | 1.82 | 6.6 | 2.06 | 1.37 | 1.29 | 1.44 |
| 2.05 | 1.42 | 1.92 | 1.28 | 2.25 | 2.07 | 1.98 | 1.2 | -1.23 | -1.44 | -1.04 | 1.3 | -1.11 | 1.4 | -1.56 | 1.21 | -1.24 | -1.48 | -2.24 | -1.5 | 1.36 | 1.61 | 1.02 | 1.45 | 1.11 | 1.22 | 1.08 | 2.94 | 1.96 | 1.12 | 1.01 | -1.89 | 2.16 | 5.95 | 1.71 | 1.64 | 1.45 | -1 |
| 2.23 | 11.19 | -1.36 | 1.14 | 1.46 | 1.3 | 1.74 | 1.23 | -1.04 | 1.34 | 1.36 | 1.08 | -1.22 | 1.14 | 2.22 | -17.78 | -1.79 | -1.89 | -1.94 | 1.04 | 1.28 | 1.19 | -1.38 | 1.19 | -1.03 | -1.23 | -1.32 | 1.72 | -2.21 | 1.46 | 1.32 | 3.93 | 2.36 | 8.06 | 2.14 | 1.66 | 1.55 | 1.24 |
| 1.36 | 2.02 | 46.02 | 12.8 | 4.62 | 4.48 | -1.56 | 1.46 | 2.11 | -14.13 | -1.39 | -6.67 | -20.15 | -3.95 | -14.58 | -5.47 | 5.03 | 353.93 | 115.27 | 1.11 | 5.2 | 3.28 | 1.91 | 1726.3 | 5222.62 | 48.83 | 9.2 | -7.06 | 2.9 | 49.79 | 19.9 | 16.44 | 26.33 | 26.65 | 9.31 | 25.87 | -1.71 | -1.71 |
| 1.52 | 6.55 | 13.23 | 7.43 | 3.22 | 2.75 | -1.82 | 1.86 | 1.84 | -7.23 | -1.48 | -7.57 | -22.2 | -3.34 | -9.06 | -4.4 | 2.44 | 343.24 | 155.86 | 1.82 | 7.06 | 3.72 | 1.66 | 1794.43 | 5308.2 | 26.06 | 8.77 | -8.84 | 1.4 | 40.92 | 18.34 | -1 | 19.05 | 29.07 | 10.69 | 27.35 | -1.83 | -1.26 |
| 1.02 | -1.07 | 33.79 | 7.26 | 5.16 | 5.65 | -1.51 | 1.86 | 1.41 | -14.76 | -2.45 | -10.91 | -21.81 | -2.8 | -7.22 | 1.37 | 4.13 | 318.27 | 97.59 | -1.11 | 5.98 | 3.89 | 1.62 | 2364.49 | 7065.44 | 35.01 | 8.59 | -8.51 | 3.74 | 41.86 | 15.48 | 1.77 | 22.62 | 26.21 | 8.86 | 32.68 | -1.63 | -1.81 |
| 1.1 | 7.37 | 12.98 | 6.45 | 3.34 | 3.56 | -1.73 | 1.9 | 1.67 | -7.7 | -1.73 | -13.1 | -23.97 | -3.43 | -2.08 | -15.78 | 2.85 | 249.85 | 112.59 | 1.41 | 5.64 | 2.88 | 1.16 | 1942.2 | 6175.41 | 23.42 | 6 | -14.55 | -1.16 | 54.43 | 20.25 | 13.16 | 24.69 | 35.54 | 11.12 | 33.11 | -1.52 | -1.46 |
| A | A | A | A | P | P | P | P | A | P | P | P | P | P | A | A | P | A | A | P | P | P | P | A | A | A | A | P | A | A | A | A | A | A | A | A | P | P |
| A | A | P | P | P | P | P | P | P | A | A | A | P | P | A | A | P | P | P | P | P | P | P | P | P | P | P | P | P | P | P | A | A | A | P | P | P | P |
| P | A | P | P | P | P | P | P | P | A | M | A | P | P | A | A | P | P | P | P | P | P | P | P | P | P | P | P | M | P | P | A | A | A | P | P | P | P |
| P | A | P | P | P | P | P | P | A | A | M | A | P | P | A | A | P | P | P | P | P | P | P | P | P | P | P | P | P | P | P | A | P | A | P | P | P | P |
| A | A | P | P | P | P | P | P | P | A | A | M | P | P | A | A | P | P | P | P | P | P | P | P | P | P | P | P | A | P | P | A | P | A | P | P | P | P |
| A | A | P | P | P | P | P | P | A | A | P | A | P | P | A | A | P | P | P | P | P | P | P | P | P | P | P | P | P | P | P | A | A | A | A | A | P | P |
| 229282_at | 210560_at | 220053_at | 210761_s_at | 201601_x_at | 214022_s_at | 201315_x_at | 218847_at | 223963_s_at | 204863_s_at | 204864_s_at | 211000_s_at | 212195_at | 212196_at | 234474_x_at | 234967_at | 205051_s_at | 206268_at | 206012_at | 205876_at | 225571_at | 225575_at | 227771_at | 219823_at | 220184_at | 230916_at | 237896_at | 231798_at | 208337_s_at | 208343_s_at | 210174_at | 1560469_at | 207742_s_at | 210391_at | 210392_x_at | 211402_x_at | 207545_s_at | 209073_s_at |
| Hs.514746 | Hs.184945 | Hs.86232 | Hs.86859 | Hs.458414 | Hs.458414 | Hs.709321 | Hs.35354 | Hs.35354 | Hs.532082 | Hs.532082 | Hs.532082 | Hs.532082 | Hs.532082 | Hs.532082 | Hs.532082 | Hs.479754 | Hs.656214 | Hs.520187 | Hs.133421 | Hs.133421 | Hs.133421 | Hs.133421 | Hs.86154 | Hs.661360 | Hs.370414 | Hs.370414 | Hs.248201 | Hs.33446 | Hs.33446 | Hs.33446 | Hs.33446 | Hs.586460 | Hs.586460 | Hs.586460 | Hs.586460 | Hs.654609 | Hs.654609 |
| GATA binding protein 6 | gastrulation brain homeobox 2 | growth differentiation factor 3 | growth factor receptor-bound protein 7 | interferon induced transmembrane protein 1 (9-27) | interferon induced transmembrane protein 1 (9-27) | interferon induced transmembrane protein 2 (1-8D) | insulin-like growth factor 2 mRNA binding protein 2 | insulin-like growth factor 2 mRNA binding protein 2 | interleukin 6 signal transducer (gp130, oncostatin M receptor) | interleukin 6 signal transducer (gp130, oncostatin M receptor) | interleukin 6 signal transducer (gp130, oncostatin M receptor) | interleukin 6 signal transducer (gp130, oncostatin M receptor) | interleukin 6 signal transducer (gp130, oncostatin M receptor) | interleukin 6 signal transducer (gp130, oncostatin M receptor) | interleukin 6 signal transducer (gp130, oncostatin M receptor) | v-kit Hardy-Zuckerman 4 feline sarcoma viral oncogene homolog | left-right determination factor 1 | left-right determination factor 2 | leukemia inhibitory factor receptor alpha | leukemia inhibitory factor receptor alpha | leukemia inhibitory factor receptor alpha | leukemia inhibitory factor receptor alpha | lin-28 homolog (C. elegans) | Nanog homeobox | nodal homolog (mouse) | nodal homolog (mouse) | noggin | nuclear receptor subfamily 5, group A, member 2 | nuclear receptor subfamily 5, group A, member 2 | nuclear receptor subfamily 5, group A, member 2 | nuclear receptor subfamily 5, group A, member 2 | nuclear receptor subfamily 6, group A, member 1 | nuclear receptor subfamily 6, group A, member 1 | nuclear receptor subfamily 6, group A, member 1 | nuclear receptor subfamily 6, group A, member 1 | numb homolog (Drosophila) | numb homolog (Drosophila) |
| GATA6 | GBX2 | GDF3 | GRB7 | IFITM1 | IFITM1 | IFITM2 | IGF2BP2 | IGF2BP2 | IL6ST | IL6ST | IL6ST | IL6ST | IL6ST | IL6ST | IL6ST | KIT | LEFTY1 | LEFTY2 | LIFR | LIFR | LIFR | LIFR | LIN28 | NANOG | NODAL | NODAL | NOG | NR5A2 | NR5A2 | NR5A2 | NR5A2 | NR6A1 | NR6A1 | NR6A1 | NR6A1 | NUMB | NUMB |

| -5.88 | -2.39 | -1.06 | 1.16 | -1.27 | 1.38 | -1.25 | -1.19 | -1.76 | -2.69 | -4.56 | -17.97 | 5.06 | -1.5 | 1.47 | 1.28 | 2.69 | 1.78 | 1.04 | 2.03 | 1.78 | -1.01 | 1.03 | -1.59 | -1.57 | -1.13 | -1.07 | -3.56 | -1.16 | -1.93 | 40.45 |
| --- | --- | --- | --- | --- | --- | --- | --- | --- | --- | --- | --- | --- | --- | --- | --- | --- | --- | --- | --- | --- | --- | --- | --- | --- | --- | --- | --- | --- | --- | --- |
| -3.68 | -3.11 | 1.09 | 1.17 | -1.49 | -1.21 | -1.1 | -4.08 | -1.87 | -3.31 | -79.11 | -5.4 | 3.33 | -1.22 | -1.11 | 1.78 | 2.04 | 1.57 | 1.16 | 1.07 | -1.88 | 1.29 | 1.15 | -1.27 | -1.01 | -1.42 | -1.04 | 1.75 | -1.71 | 1.47 | 27 |
| -28.37 | -3.39 | 1.23 | 1.42 | -1.13 | 1.26 | -1.44 | -8.24 | -1.93 | -2.16 | -3.47 | -6.54 | 5.45 | -1.18 | 1.3 | 1.3 | 2.19 | 1.36 | 1.37 | 2.49 | -1.32 | 1.37 | 1.5 | 1.07 | 1.28 | -1.37 | -1.32 | 2.58 | 2.31 | -3.98 | 17.4 |
| -3.55 | -3 | 1.05 | 1.19 | -1.49 | -1.29 | -1.42 | -4.93 | -2.11 | -32.64 | -9.06 | -18.12 | 2.27 | -1.1 | 1.28 | 1.62 | 2.58 | 1.8 | 1.09 | -1.36 | -1.39 | 1.24 | 1.13 | 1.12 | -2.8 | -1.36 | -1.07 | -1.64 | -3.18 | 16.53 | 390.9 |
| -1.64 | 6.12 | 10.15 | 371.69 | -1.49 | -1.29 | -1.93 | 3.1 | -3.71 | 1.1 | 3.73 | -1.13 | 2.92 | 1.4 | -3.46 | -3.44 | 1387.88 | 2439.08 | 52.69 | 45.28 | 4.3 | 11136.52 | 2488 | 2.07 | 1.47 | 1.93 | 4.15 | 1.23 | 5.68 | -67.52 | -19.33 |
| -1.02 | 4.7 | 11.72 | 374.1 | -1.76 | -2.15 | -1.7 | -1.11 | -3.95 | -1.12 | -4.66 | 2.95 | 1.92 | 1.73 | -5.66 | -2.47 | 1052 | 2154.1 | 58.33 | 23.95 | 1.28 | 14447.55 | 2778.67 | 2.59 | 2.28 | 1.54 | 4.26 | 7.69 | 3.87 | -23.79 | -28.95 |
| -7.9 | 4.32 | 13.21 | 452.28 | -1.34 | -1.41 | -2.23 | -2.24 | -4.07 | 1.37 | 4.9 | 2.44 | 3.14 | 1.78 | -3.92 | -3.38 | 1130.4 | 1861.07 | 69.21 | 55.44 | 1.83 | 15425.7 | 3627.56 | 3.54 | 2.96 | 1.59 | 3.34 | 11.3 | 15.23 | -139.56 | -44.93 |
| 1.01 | 4.87 | 11.27 | 379.87 | -1.75 | -2.29 | -2.18 | -1.34 | -4.47 | -11.06 | 1.87 | -1.14 | 1.31 | 1.91 | -3.96 | -2.71 | 1333.58 | 2470.2 | 55.25 | 16.43 | 1.73 | 13960.44 | 2723.31 | 3.69 | -1.21 | 1.61 | 4.12 | 2.68 | 2.08 | -2.12 | -2 |
| P | A | P | A | P | P | P | A | P | A | A | A | A | P | P | P | A | A | A | A | A | A | A | P | A | P | A | A | A | P | P |
| A | A | P | P | P | P | P | A | P | A | A | A | A | P | P | P | P | P | P | A | P | P | P | P | A | P | P | A | P | A | A |
| A | P | P | P | P | P | P | A | P | A | A | A | A | P | P | P | P | P | P | A | P | P | P | P | P | P | P | A | P | P | A |
| A | A | P | P | P | P | P | A | P | A | P | A | A | P | P | P | P | P | P | M | A | P | P | P | P | P | P | A | P | A | A |
| A | A | P | P | P | P | P | A | P | A | A | A | A | P | P | P | P | P | P | A | P | P | P | P | A | P | P | A | P | P | P |
| P | P | P | P | P | P | P | A | P | A | P | A | A | P | P | P | P | P | P | A | A | P | P | P | M | P | P | A | P | P | A |
| 230462_at | 236930_at | 201578_at | 208286_x_at | 204053_x_at | 204054_at | 211711_s_at | 222176_at | 225363_at | 233254_x_at | 233314_at | 242622_x_at | 204535_s_at | 212920_at | 206805_at | 244163_at | 223121_s_at | 223122_s_at | 213721_at | 213722_at | 214178_s_at | 228038_at | 206286_s_at | 207199_at | 1555271_a_at | 219735_s_at | 227642_at | 229341_at | 208275_x_at | 214218_s_at | 221728_x_at |
| Hs.654609 | Hs.654609 | Hs.690098 | Hs.450254 | Hs.500466 | Hs.500466 | Hs.500466 | Hs.500466 | Hs.500466 | Hs.500466 | Hs.500466 | Hs.500466 | Hs.631513 | Hs.631513 | Hs.252451 | Hs.252451 | Hs.481022 | Hs.481022 | Hs.518438 | Hs.518438 | Hs.518438 | Hs.518438 | Hs.385870 | Hs.492203 | Hs.492203 | Hs.156471 | Hs.156471 | Hs.156471 | Hs.458406 | Hs.529901 | Hs.529901 |
| numb homolog (Drosophila) | Numb homolog (Drosophila) | podocalyxin-like | POU class 5 homeobox 1 POU class 5 homeobox 1B POU class 5 homeobox 1 pseudogene 3 POU class 5 homeobox 1 pseudogene 4 | phosphatase and tensin homolog | phosphatase and tensin homolog | phosphatase and tensin homolog | phosphatase and tensin homolog | phosphatase and tensin homolog | phosphatase and tensin homolog | phosphatase and tensin homolog | Phosphatase and tensin homolog | RE1-silencing transcription factor | RE1-silencing transcription factor | sema domain, immunoglobulin domain (Ig), short basic domain, secreted, (semaphorin) 3A | sema domain, immunoglobulin domain (Ig), short basic domain, secreted, (semaphorin) 3A | secreted frizzled-related protein 2 | secreted frizzled-related protein 2 | SRY (sex determining region Y)-box 2 | SRY (sex determining region Y)-box 2 | SRY (sex determining region Y)-box 2 | SRY (sex determining region Y)-box 2 | teratocarcinoma-derived growth factor 1 teratocarcinoma-derived growth factor 3, pseudogene | telomerase reverse transcriptase | telomerase reverse transcriptase | transcription factor CP2-like 1 | transcription factor CP2-like 1 | transcription factor CP2-like 1 | undifferentiated embryonic cell transcription factor 1 | X (inactive)-specific transcript (non-protein coding) | X (inactive)-specific transcript (non-protein coding) |
| NUMB | NUMB | PODXL | POU5F1  POU5F1B POU5F1P3 POU5F1P4 | PTEN | PTEN | PTEN | PTEN | PTEN | PTEN | PTEN | PTEN | REST | REST | SEMA3A | SEMA3A | SFRP2 | SFRP2 | SOX2 | SOX2 | SOX2 | SOX2 | TDGF1 TDGF3 | TERT | TERT | TFCP2L1 | TFCP2L1 | TFCP2L1 | UTF1 | XIST | XIST |

| -2.49 | -2.76 | -1.12 | -2.37 | -2.25 | -1.25 | -1.39 | 1.83 | 2.19 | *, Flag indicates whether a transcript is Present (P), Marginal (M) or Absent (A), which were assigned by the Detection Algorithm. |
| --- | --- | --- | --- | --- | --- | --- | --- | --- | --- |
| 4.53 | 1.62 | 1.71 | 1.29 | 1.37 | -1.75 | -1.08 | -1.18 | 1.73 |
| -5.55 | 1.38 | -5.16 | -2.59 | -6.58 | -1.44 | 1.06 | 2.07 | 2.32 |
| 107.85 | 2.54 | 2.78 | 16.08 | -3.48 | -2.63 | 1.13 | -1.39 | 1.91 |
| -606.57 | -33.87 | -68.88 | -74.36 | 1.03 | 1.24 | 1325.95 | 989.65 | 239.15 |
| -53.77 | -7.57 | -35.96 | -24.28 | 3.18 | -1.13 | 1701.83 | 458.49 | 188.73 |
| -1352.88 | -8.92 | -316.91 | -81.32 | -2.83 | 1.07 | 1954.03 | 1119.96 | 253 |
| -2.26 | -4.84 | -22.04 | -1.95 | -1.5 | -1.7 | 2068.05 | 389.51 | 208.11 |
| P | P | P | P | A | P | A | A | A |
| A | A | A | A | A | A | P | P | P |
| A | A | A | P | A | P | P | P | P |
| A | A | A | A | A | A | P | P | P |
| P | A | P | P | A | A | P | P | P |
| A | A | A | A | A | A | P | P | P |
| 224588_at | 224589_at | 224590_at | 227671_at | 235446_at | 243712_at | 243161_x_at | 1554776_at | 1554777_at |
| Hs.529901 | Hs.529901 | Hs.529901 | Hs.529901 | Hs.724376 | Hs.529901 | Hs.335787 | Hs.335787 | Hs.335787 |
| X (inactive)-specific transcript (non-protein coding) | X (inactive)-specific transcript (non-protein coding) | X (inactive)-specific transcript (non-protein coding) | X (inactive)-specific transcript (non-protein coding) | X (inactive)-specific transcript (non-protein coding) | X (inactive)-specific transcript (non-protein coding) | zinc finger protein 42 homolog (mouse) | zinc finger protein 42 homolog (mouse) | zinc finger protein 42 homolog (mouse) |
| XIST | XIST | XIST | XIST | XIST | XIST | ZFP42 | ZFP42 | ZFP42 |
