# Supplementary material for: Feeder-Free Generation and Long-Term Culture of Human Induced Pluripotent Stem Cells Using Pericellular Matrix of Decidua Derived Mesenchymal Cells
Source: PLoS One. 2013 Jan 31;8(1):e55226. doi: 10.1371/journal.pone.0055226 (PMC3561375; doi:10.1371/journal.pone.0055226)
Supplement: Table S5 — Reprogramming efficiencies. (DOC) [file pone.0055226.s006.doc]

**Table S5: Reprogramming efficiencies**

| Parental cells | Substrate | Medium | Induced cell  number | 1st | | 2nd | |
| --- | --- | --- | --- | --- | --- | --- | --- |
|  |  |  |  | Number of ES-like colonies | Reprogramming efficiency (%) | Number of ES-like colonies | Reprogramming efficiency (%) |
| DMC71 | MEF | hESC | 60000 | 6 | 0.010 | 1 | 0.0017 |
|  | PCM-DM |  | 60000 | - | - | 4 | 0.0067 |
|  | Matrigel |  | 60000 | - | - | 2 | 0.0033 |
|  | Gelatin |  | 60000 | - | - | 0 | 0 |
|  | PCM-DM | MEF-CM | 60000 | 1 | 0.0017 | 8 | 0.0133 |
|  | Matrigel |  | 60000 | 21 | 0.0350 | 7 | 0.0117 |
|  | Gelatin |  | 60000 | 1 | 0.0017 | 3 | 0.0050 |
| DMC72 | MEF | hESC | 60000 | 1 | 0.0017 | 1 | 0.0017 |
|  | PCM-DM |  | 60000 | - | - | 2 | 0.0033 |
|  | Matrigel |  | 60000 | - | - | 0 | 0 |
|  | Gelatin |  | 60000 | - | - | 0 | 0 |
|  | PCM-DM | MEF-CM | 60000 | 5 | 0.0083 | 3 | 0.0050 |
|  | Matrigel |  | 60000 | 33 | 0.0550 | 9 | 0.0150 |
|  | Gelatin |  | 60000 | 5 | 0.0083 | 0 | 0 |
| DMC75 | MEF | hESC | 60000 | 5 | 0.0083 | 1 | 0.0017 |
|  | PCM-DM |  | 60000 | - | - | 3 | 0.0050 |
|  | Matrigel |  | 60000 | - | - | 0 | 0 |
|  | Gelatin |  | 60000 | - | - | 0 | 0 |
|  | PCM-DM | MEF-CM | 60000 | 21 | 0.0350 | 33 | 0.0550 |
|  | Matrigel |  | 60000 | 74 | 0.1233 | 87 | 0.1450 |
|  | Gelatin |  | 60000 | 10 | 0.0167 | 18 | 0.0300 |
| DMC76 | MEF | hESC | 60000 | - | - | 1 | 0.0017 |
|  | PCM-DM |  | 60000 | - | - | 10 | 0.0167 |
|  | Matrigel |  | 60000 | - | - | 5 | 0.0083 |
|  | Gelatin |  | 60000 | - | - | 0 | 0 |
|  | PCM-DM | MEF-CM | 60000 | - | - | 54 | 0.0900 |
|  | Matrigel |  | 60000 | - | - | 61 | 0.1017 |
|  | Gelatin |  | 60000 | - | - | 20 | 0.0333 |
| DMC85 | MEF | hESC | 60000 | - | - | 1 | 0.0017 |
|  | PCM-DM |  | 60000 | - | - | 1 | 0.0017 |
|  | Matrigel |  | 60000 | - | - | 0 | 0 |
|  | Gelatin |  | 60000 | - | - | 0 | 0 |
|  | PCM-DM | MEF-CM | 60000 | - | - | 11 | 0.0183 |
|  | Matrigel |  | 60000 | - | - | 10 | 0.0167 |
|  | Gelatin |  | 60000 | - | - | 0 | 0 |
| DMC92 | MEF | hESC | 60000 | - | - | 1 | 0.0017 |
|  | PCM-DM |  | 60000 | - | - | 0 | 0 |
|  | Matrigel |  | 60000 | - | - | 0 | 0 |
|  | Gelatin |  | 60000 | - | - | 0 | 0 |
|  | PCM-DM | MEF-CM | 60000 | - | - | 1 | 0.0017 |
|  | Matrigel |  | 60000 | - | - | 10 | 0.0167 |
|  | Gelatin |  | 60000 | - | - | 1 | 0.0017 |
